# Quantitative analysis of macroscopic solute transport in the murine brain

**DOI:** 10.1186/s12987-021-00290-z

**Published:** 2021-12-07

**Authors:** Lori A. Ray, Martin Pike, Matthew Simon, Jeffrey J. Iliff, Jeffrey J. Heys

**Affiliations:** 1 Department of Chemical and Biological Engineering, Montana State University, Bozeman, USA; 2 Advanced Imaging Research Center, Oregon Health and Sciences University, Portland, USA; 3 Department of Anesthesiology and Perioperative Medicine, Oregon Health and Science University, Portland, USA; 4 Neuroscience Graduate Program, Oregon Health and Science University, Portland, USA; 5 VISN 20 Mental Illness Research, Education and Clinical Center (MIRECC), VA Puget Sound Health Care System, Seattle, USA; 6 Department of Psychiatry and Behavioral Sciences, University of Washington School of Medicine, Seattle, USA; 7 Department of Neurology, University of Washington School of Medicine, Seattle, USA; 8 Present Address: Denali Therapeutics, San Francisco, USA

**Keywords:** Biotransport, Brain transport, Glymphatic, Perivascular transport, Interstitial transport, Dynamic contrast-enhanced MRI

## Abstract

**Background:**

Understanding molecular transport in the brain is critical to care and prevention of neurological disease and injury. A key question is whether transport occurs primarily by diffusion, or also by convection or dispersion. Dynamic contrast-enhanced (DCE-MRI) experiments have long reported solute transport in the brain that appears to be faster than diffusion alone, but this transport rate has not been quantified to a physically relevant value that can be compared to known diffusive rates of tracers.

**Methods:**

In this work, DCE-MRI experimental data is analyzed using subject-specific finite-element models to quantify transport in different anatomical regions across the whole mouse brain. The set of regional effective diffusivities ($D_{eff}$), a transport parameter combining all mechanisms of transport, that best represent the experimental data are determined and compared to apparent diffusivity ($D_{app}$), the known rate of diffusion through brain tissue, to draw conclusions about dominant transport mechanisms in each region.

**Results:**

In the perivascular regions of major arteries, $D_{eff}$ for gadoteridol (550 Da) was over 10,000 times greater than $D_{app}$. In the brain tissue, constituting interstitial space and the perivascular space of smaller blood vessels, $D_{eff}$ was 10–25 times greater than $D_{app}$.

**Conclusions:**

The analysis concludes that convection is present throughout the brain. Convection is dominant in the perivascular space of major surface and branching arteries (*Pe* > 1000) and significant to large molecules (> 1 kDa) in the combined interstitial space and perivascular space of smaller vessels (not resolved by DCE-MRI). Importantly, this work supports perivascular convection along penetrating blood vessels.

**Supplementary Information:**

The online version contains supplementary material available at 10.1186/s12987-021-00290-z.

## Background

Molecular transport is an essential element in physiological brain function, contributing to neurotransmission, ion homeostasis, nutrient delivery and waste clearance [1]. Changes in interstitial molecular transport have been implicated in several pathological states, including the proposal that impairment of interstitial peptide and protein clearance may underlie the vulnerability of the aging or injured brain to the development of protein aggregates in neurodegenerative disease [2–4].

While solute transport within the brain parenchyma is classically attributed to diffusion [5], Rennels et al. [6] and Cserr et al. [7] reported that tracers injected both into the brain interstitium and the cerebrospinal fluid compartments move preferentially along perivascular spaces (PVSs) surrounding the cerebral vasculature and that the observed tracer movement was too fast to be explained by diffusion alone. These PVSs are annular spaces bounded by the vascular wall on the inside and by perivascular astroglial endfeet on the outside. Patterns of tracer distribution demonstrate that PVSs are continuous both with the brain interstitium and the cerebrospinal fluid (CSF) surrounding the brain.

**Fig. 1:**
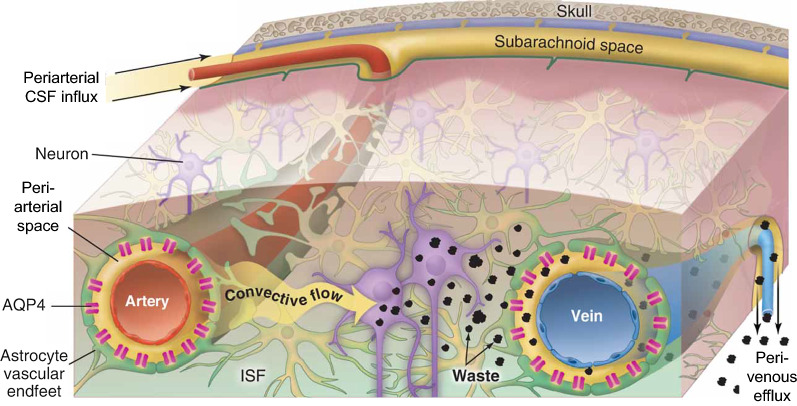
Perivascular glymphatic transport in the brain. Schematic depicting the proposed mechanism underlying molecular transport in brain tissue, the glymphatic system. Perivascular spaces (PVS) surround cerebral blood vessels and are bounded by the vascular wall on the inside and by the ‘endfeet’ of astrocyte cells (green) on the outside. It is proposed that cerebrospinal fluid moves inward from the subarachnoid space along periarterial spaces surrounding penetrating arteries deep into the brain. The fluid enters the interstitium through gaps between astroglial endfeet. Interstitial solutes (‘waste’) are cleared by interstitial convection towards peri-venous spaces and extracranial efflux routes. Perivascular CSF influx and interstitial solute efflux is facilitated by astroglial aquaporin-4 (AQP4) water channels. Reproduced from [13] with nomenclature updated from ‘para-arterial’ to ‘periarterial’

This topic has risen in visibility over recent years with the description of the ‘glymphatic’ system [3, 8–10]. The glymphatic system is a brain-wide system for molecular transport (Fig. 1) where: (1) subarachnoid CSF enters the brain along PVS surrounding penetrating arteries (moving in the same direction as blood flow), (2) fluid and solutes exchange into the interstitial space through gaps between astroglial endfeet, and (3) interstitial fluid and solutes drain along PVSs surrounding large-caliber draining veins towards sinus-associated CSF compartments. The subsequent characterization of meningeal lymphatic vessels [11, 12] associated with dural sinuses provided a final potential step in the clearance of these interstitial solutes from the cranium along the lymphatic drainage. In the glymphatic model, PVSs surrounding the branching cerebral vasculature provide a low-resistance conduit for efficient exchange of fluid between the brain surface and regions deep within the brain, supporting solute delivery and waste clearance.

The existence of both perivascular and interstitial convection in the brain has undergone much debate [14–22]. Periarterial flow has been measured directly at 1.2 mm/min [18, 19] and entrains to arterial pulsation [9, 18, 23], suggesting that peristaltic flow within the PVS is generated by pulsation of the arterial (inner) wall against a mostly rigid outer ‘wall’ comprised of ensheathing leptomeninges or astroglial endfeet. However, to date mathematical and computational models of arterial-pulse driven periarterial flow do not replicate the flows observed experimentally [20, 24–27], concluding a physiologically reasonable, but as yet unidentified, static pressure gradient is required to generate flows observed experimentally [26, 27]. Dispersion has also been predicted to moderately enhance periarterial transport over diffusion alone [20, 28]. Dispersion is a transport effect caused by the coupling of concentration gradients and fluid flow [29]. Periarterial dispersion is caused by fluctuations in flow generated by arterial pulsation that facilitates mixing and accelerate transport relative to diffusion alone or the net flow of the fluid.

Measurements of periarterial flow have been conducted only along distil segments of the middle cerebral artery (MCA) on the brain surface [18, 19]. Observed flow characteristics are indicative of flow in an open channel [18, 23], which has a lower hydraulic resistance than a porous, media-filled space. However, the 1-μm diameter microspheres tracked in the experiments did not enter PVSs surrounding penetrating arteries [19, 23]. It is possible that periarterial flow may only occur around surface arteries [19] or that periarterial space surrounding penetrating vessels may be “somewhat porous”, excluding the relatively large microspheres while permitting the transport of large soluble tracers [8, 19, 23].

In the interstitial space, transport models have estimated interstitial flow superficial velocity ranging from no flow to 0.05 mm/min [30–32]. An interstitial flow of 0.01 mm/min is sufficient to affect the transport rate of macromolecules greater than 1 kDa in size, including the peptides and proteins (amyloid β, tau, a synuclein) implicated in neurodegenerative diseases [30, 32]. Differences between models of interstitial flow originate from varying assumptions about the hydraulic conductivity of brain tissue (the ease with which fluid can move through ‘porous’ interstitial space) and pressure gradients between periarterial and perivenous spaces, which are not known. With these uncertainties surrounding the existence, magnitude and extent of perivascular and interstitial flow, it is unclear whether convection is a significant contributor to the transport of molecules within brain tissue.

Dynamic contrast-enhanced magnetic resonance imaging (DCE-MRI) has emerged as the gold-standard for investigating molecular transport within the healthy and diseased rodent and human brain, with a recent review summarizing fifteen such studies since the first experiment in 2013 [33, 34]. DCE-MRI offers a brain-wide, macroscopic view of transport, complimentary to the microscopic view of flow surrounding single vessels provided by 2-photon microscopy [8, 18]. Studies utilizing DCE-MRI demonstrate that contrast agents injected into the subarachnoid CSF of the murine brain move along preferential pathways following major arteries near the surface of the brain, then penetrate into the brain at a rate too rapid to be described by diffusion alone [34–40]. Typically, cluster analysis is performed on time-series signal data to show the speed of contrast agent uptake in different brain regions or the general direction of contrast agent movement [17, 34, 41]. Optimal mass transport (OMT) modelling has emerged as an excellent tool for visualizing movement of intrathecal contrast agents through the brain [42]. However, these semi-quantitative techniques do not permit the determination of the fundamental parameters characterizing molecular transport that are independent of experimental procedures and allow direct comparison with known diffusion rates. Within these DCE-MRI studies, measured signal changes have been regarded as a surrogate for contrast concentration, assuming a direct proportionality between concentration and signal change, when this relationship is somewhat more complex (see “Methods”).

Previous computational models of brain transport predicting fundamental parameters have been at the microscale and have only qualitatively compared results to experimental data [20, 30, 31]. An exception is Valnes et al., who developed a finite-element model simulating contrast transport in a section of the human brain [43], utilizing DCE-MRI data derived from human studies [44, 45] to estimate transport parameters in grey and white matter. However, the complexity of the highly-folded surface of the human brain, variability among study participants, and the noisy nature of DCE-MRI data resulted in dependence of results upon boundary-data filtering methods. Here, a finite-element transport model is developed for the simpler case of the mouse brain. The mouse brain has a smoother cortical surface, little white matter, and smaller size that enables modelling of the whole brain while small-animal MRI has the resolution needed to identify the major cerebral vasculature. This permits the quantification of transport along major periarterial conduits, which are important to molecular movement across the brain.

In this study, a simplified finite-element model of transport in the brain is applied to analyze DCE-MRI datasets and determine fundamental parameters describing macroscopic transport mechanisms across the brain volume. By analyzing the physically relevant variable of concentration (calculated from DCE-MRI signal), using subject-specific models segmented based on anatomical landmarks and concentration dynamics, and applying theory from transport phenomena, we aim to calculate transport parameters that can be directly compared to known diffusivity through brain tissue ($D_{app}$) and are independent of the experimental situation. Our analysis shows evidence of: (1) convection along preferential routes surrounding major arteries that is consistent with experimental measurements of periarterial flow, and (2) convection at a slower, but significant rate through the brain tissue, which includes smaller perivascular and interstitial space.

## Methods

The purpose of this work is to analyze DCE-MRI data using theories from mass transport to quantify experimentally observed rates of macroscopic solute transport, i.e. at the scale of the whole brain, that can be compared between experiments and directly compared to known rates of diffusion. Through comparison of overall transport rates to known diffusive rates, and using current understandings of brain transport from the literature, conclusions can be drawn about possible modes of transport in different anatomical regions. This approach provides insight into the properties of glymphatic transport, including whether convective fluid flow is an important contributor to molecular transport within brain tissue.

### Methodology

Transport of molecules in brain tissue may occur by a combination of mechanisms according to Eq. 1, including diffusion, dispersion, convection, and source or sink terms (given in order on the right-hand side of Eq. 3):

1 $$\frac{\partial c}{{\partial t}} = D_{app} \nabla^{2} c{ } + D_{disp} \nabla^{2} c - v \cdot \nabla c + s\left( c \right) - f\left( c \right)$$

where *c* = concentration, $D_{app}$ = apparent diffusivity, $D_{disp}$ = dispersion coefficient, *v* = superficial velocity (a vector field), $s\left( c \right)$  = spatially dependent source term (e.g., injection), and $f\left( c \right)$  = sink term (e.g., cellular uptake, adsorption, efflux route). The contrast agent was chosen for its lack of biological activity in the CNS, therefore cellular uptake and adsorption can be ignored. For a biologically active molecule, transport would be slowed by these processes of interaction with cells.

The convective term in Eq. (1) presents a challenge when characterizing macroscopic transport and utilizing data with a resolution of only 100 μm, which does not resolve the microvessels that provide the primary conduit for fluid transport throughout the brain. The fluid velocity (*v*) in the convective term is a vector, meaning it has both magnitude and direction. Fluid velocity, as described by the glymphatic theory, may occur in several directions within a single voxel—first along periarterial space, which is branching in nature, then across interstitial space from periarterial to perivenous, likely to be perpendicular to the periarterial flow, and then along perivenous space, which is also branching in nature. Therefore, fluid velocity observed at the resolution of an MRI voxel represents a combination of these velocity mechanisms resulting in a net propagation of the contrast agent across the voxel more rapid than diffusion alone.

Despite the multi-directional fluid velocities within a voxel, is it possible to determine a “net” velocity magnitude within an anatomical region using the DCE-MRI data and a transport model built from Eq. 1? Characterizing transport using this approach would still require a unit vector describing the direction of the velocity for each mesh element. As fluid moves through the brain, following the branching network of the vasculature and crossing interstitial space, it changes direction, likely element-by-element. In addition, MRI resolution allows identification of large caliber vessels, but contains no information about the smaller vasculature that might inform unit vector assumptions. Such an approach would require unreasonable assumptions about velocity direction, adding a great deal of uncertainty to the calculations.

Is it feasible to build a model from first principles to predict the details of glymphatic flow across the whole brain that can be compared to the DCE-MRI data? Fluid velocity, according to the glymphatic theory, is driven by mechanisms at the microscopic level with the primary driver being periarterial flow. As described in the introduction, experimental evidence supports periarterial flow entrained with arterial pulsation, however observed periarterial flows [18, 19] have not yet been predicted from first principles assuming this mechanism [20, 24, 26, 27]. In fact, using the most sophisticated computational models to date, both Daversin-Catty et al. and Kedarasetti et al. demonstrated arterial pulsation alone produces negligible net flow and a static pressure gradient of about 2 mmHg/m (or 0.1 mmHg along the 5 mm MCA) is required to achieve experimentally observed periarterial flows [26, 27]. (An elliptical periarterial cross section, as observed experimentally [18], produces half the hydraulic resistance of the circular cross sction [46] used in the computational models, which would further reduce the required pressure gradient by as much as half). Although the cardiac cycle produces a static pressure gradient of this magnitude, it is not clear how that might be transmitted to the fluid in the periarterial space [27]. Others have argued that hydrostatic pressure gradients within the brain are not sufficient to generate observed flows [47]. Interstitial flow has been shown to be well described by Darcy’s law for flow through porous media ($v = - k\nabla P$). However, the periarterial pressures that drive both interstitial and perivenous flow are unknown. Given the complexity of the vascular network across the brain and current limitations of first-principles models for periarterial flow, it is not currently feasible to predict fluid flow throughout the brain.

Provided the above limitations on calculation or estimation of fluid velocity at the macroscopic scale, an overall transport parameter approach is utilized:

2 $$\frac{\partial c}{{\partial t}} = D_{eff} \nabla c^{2} + s\left( c \right)$$

where all transport mechanisms are ‘lumped’ into a single parameter, effective diffusivity ($D_{eff}$) and this overall transport parameter can be compared to the known diffusivity of the contrast agent to draw conclusions about additional transport mechanisms. Effective diffusivity, $D_{eff}$, represents a composite of diffusion, perivascular dispersion, perivascular convection, and interstitial convection within a single transport parameter. Modelling transport with $D_{eff}$ under conditions where the dominant mechanisms are uncertain is a general approach that has been taken previously [43] to quantify and compare transport rates within the CNS. The downside of this simplification is that it forces the mathematical form of diffusion, and all mechanisms of transport are lumped into a single parameter. The benefits of the methodology are (1) the resulting problem is relatively simple and solvable in a complex geometry with few assumptions, (2) it requires only concentration data, obtained directly from DCE-MRI, and (3) the result is directly comparable to the known diffusivity of the contrast agent. Being agnostic towards the mechanism of transport is advantageous given the broad range of models and results surrounding the subject of brain transport, spanning from diffusion, to dispersion, to convection [25]. Thus, given real uncertainty about the presence and driving forces of convective flow in the brain, the simplified model is well-suited for impartial characterization of brain transport measured by DCE-MRI.

The $D_{eff}$ values for each segmented anatomical volume are compared to the known apparent diffusivity ($D_{app}$) of gadoteridol:

3 $$D_{app} = D/\lambda^{2} = 0.016/1.73^{2} = 0.005\, {\text{mm}}^{2} /{\text{min}}$$

where *D* = 0.016 mm^2^/min [48] is the free diffusivity of gadoteridol, and λ = 1.6 [49] or λ = 1.85 [32] is tortuosity, which represents the degree to which molecular transport is slowed by the porous medium. From these comparisons, we can infer the prevalence and magnitude of convection and dispersion for each anatomical subdomain in the transport model. For example, if significant convection is present, the $D_{eff}$ values will be orders of magnitude greater than the $D_{app}$ values.

### Method overview

**Fig. 2:**
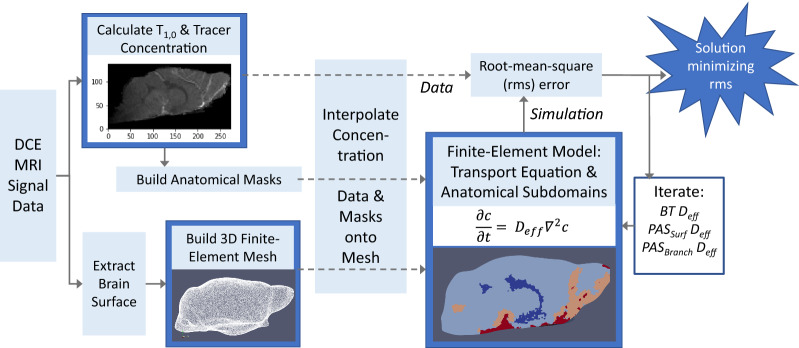
Schematic of brain transport analysis using finite-element modeling with DCE-MRI Data. DCE-MRI signal data is used to calculate contrast agent concentration and $T_{1,0}$, a parameter used to identify different types of tissues. Contrast concentration and $T_{1,0}$ are used to define anatomical masks for regions relevant to brain-wide transport, including the ventricular system, the macroscopic arterial vasculature, and its associated perivascular spaces. DCE-MRI data is also utilized to extract the surface of the brain. The anatomical masks are unique to each experimental subject. A 3D tetrahedral mesh is built from the brain surface. The concentration data and anatomical masks are interpolated onto the mesh. A finite-element model is developed utilizing the simplified mass transport equation (Eq. 2) and subdomains defined by the anatomical masks (Fig. 3). Unique effective diffusivities are applied to each subdomain. Simulations are performed for varying effective diffusivities to find the combination of effective diffusivities that minimizes the difference between the concentrations data and the simulation

An overview of the process used to estimate transport parameters for different anatomical regions using finite-element modelling and DCE-MRI data is presented in Fig. 2. At a high level, DCE-MRI signal data are used to calculate contrast agent concentration and to define discrete anatomical regions for the computational model relevant to brain-wide transport. Subject-specific, finite-element models of molecular transport are built for the whole mouse brain where unique transport parameters are applied to each defined anatomical region. Transport parameter sets are varied, and simulations performed to determine the optimal combination of parameters for each subject that minimizes the difference between the concentration data and the simulation. Calculated transport parameters are then compared to the known diffusivity of the contrast agent through brain tissue to draw conclusions about convective transport in the different regions of the brain.

**Fig. 3:**
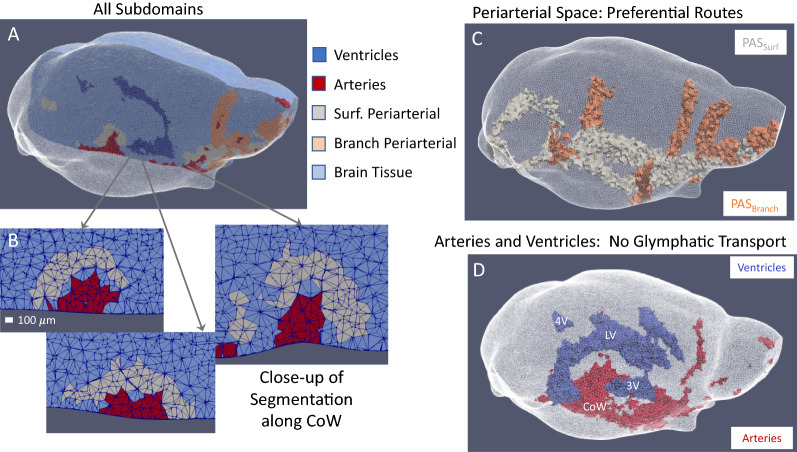
Anatomical details extracted from MRI data define transport model subdomains. Structure MRI scans and DCE-MRI data were used to build subdomains within the whole-brain finite-element model. Data were analyzed to determine transport parameters for each subdomain within the whole-brain model. **A** Subject-specific, 3D tetrahedral mesh of mouse brain in translucent white (crystal brain). Sagittal cross-section shows all five anatomical subdomains within the finite-element model. **B** Close up of segmentation between artery and periarterial space as 2D coronal slices (perpendicular to cross-section shown in (**A**)) along the left branch of the Circle of Willis, where lines depict the edges of tetrahedral mesh vertices. The width of the periarterial space ranges from three to nine vertices. Because the periarterial space is defined primarily by concentration, sometimes a buffer one or two vertices thick resides between the artery and the periarterial space that is indicative of boundaries between regions where one or more materials may reside in the same voxel and influence the measured signal (see “Sources of Error”). The artery, which holds no contrast agent, exhibits no post-contrast signal change, while the periarterial space, which has high contrast concentration, exhibits large post-contrast signal change. The intermediate region is a combination of both, which produces a signal in between thus excluding the voxel from the definitions of either the arterial or periarterial region. **C** 3D ‘crystal brain’ image of the surface (light grey) and branching (peach) periarterial subdomains. Translucent white shows the surface of the brain. **D** 3D ‘crystal brain’ image of arterial (red) and ventricular (blue) subdomains (lateral ventricle (LV), Third Ventricle (3 V), Fourth Ventricle (4 V), Circle of Willis (CoW))

### DCE-MRI experiments

The present study reflects an analysis of previously published DCE-MRI data collected in n = 4 male and female 3–6 month old C57 Bl/6 mice [17]. Briefly, ketamine/xylazine-anesthetized (100/10 mg/kg, respectively) animals were injected with 10 μl of 68 mM gadoteridol contrast agent (550 Da) at a rate of 0.5 µl/min via a pulled glass pipette surgically inserted through the atlanto-occipital membrane into the aqueduct between the fourth ventricle and the subarachnoid space (SAS). Following contrast agent infusion, a 2 µl chase of saline was infused. Serial 3D FLASH T1-weighted MR images with isotropic (100 × 100 × 100 µm) voxels were obtained at 11.75 T at 10-min intervals over 80 min with the mouse in the prone position. The image series for each subject was aligned to a baseline image using a linear rigid-body registration, followed by masking to remove non-brain regions (see Additional file 2), and subsequent linear registration to the Badhwar mouse hippocampal Atlas with 60 × 60 × 60 µm isotropic voxels (FSL). Due to the high magnetic field utilized in this experiment, interference of T2/T2* effects in T1-weighted images is encountered in places of high contrast concentration and in proximity to bones like the skull. These magnetic susceptibility effects cause any contrast enhancement to be obscured by a decreased intensity in the affected voxels.

As reported in our prior study, early contrast enhancement occurred along the ventral surface of the brain, after which parenchymal enhancement throughout brain tissue began to increase with high concentrations observed in the olfactory bulb, a known glymphatic efflux route.

### Calculation of contrast concentration and identification of anatomical regions

To quantify transport parameters, concentration–a fundamental physical variable–is required. The majority of DCE-MRI data in the literature are reported and analyzed as signal. Measured signal changes are often regarded as a surrogate for contrast concentration, assuming direct proportionality. T1-weighted signal is increased by the presence of the contrast agent, but the relationship between signal change and concentration is more complex. Gadoteridol concentration is related to MRI signal by [50]:

4 $$\left[ {gadoteridol} \right]{ } \cong \frac{1}{{r_{1} \cdot T_{1,0} }} \left( {\frac{{S_{Gd} - S_{0} }}{{S_{0} }}} \right)$$

where *r*_1_ = Gadoteridol (Prohance) relaxivity, 3.2 × 10^–3^ L/mmol-ms [51]. $T_{1,0}$ = pre-contrast relaxation time (ms). $S_{Gd}$ = signal intensity after contrast injection (as a function of time, dimensionless). $S_{0}$  = baseline signal intensity prior to contrast agent injection (dimensionless).

The proportionality assumption between contrast concentration and signal change is correct if $T_{1,0}$ is constant across the sample. However, $T_{1,0}$ is dependent on the molecular environment and varies significantly (500–4500) across different tissues in a biological subject, especially one as complex as the brain. $T_{1,0}$ can be estimated from baseline MRI signals collected at different flip angles according to the following equation [52]:

5 $$S\left( {M_{0} ,{ }\alpha } \right) = { }M_{0} {\text{ sin}}\left( \alpha \right){ }\frac{{1 - e^{{{\raise0.7ex\hbox{${ - TR}$} \!\mathord{\left/ {\vphantom {{ - TR} {T_{1,0} }}}\right.\kern-\nulldelimiterspace} \!\lower0.7ex\hbox{${T_{1,0} }$}}}} }}{{1 - cos\left( \alpha \right){ }e^{{{\raise0.7ex\hbox{${ - TR}$} \!\mathord{\left/ {\vphantom {{ - TR} {T_{1,0} }}}\right.\kern-\nulldelimiterspace} \!\lower0.7ex\hbox{${T_{1,0} }$}}}} }}$$

where *TR* = repetition time (16 ms for the experiments reported here), and α = flip angle.

For each voxel, $T_{1,0}$ is calculated from $S_{0}$ (baseline) images at α= 3° and 15° using a curve fit to Eq. 5. Knowing $T_{1,0}$, Eq. 4 is used to calculate gadoteridol concentration from DCE-MRI signal for each voxel and time point. The calculated concentration, [gadoteridol], is a superficial concentration ($\overline{c}$) used in porous media theory, where $\overline{c} = c \cdot \phi$ and *∅* is void fraction or porosity. It is assumed the void fraction is constant across the brain; *∅* has a well-known value of approximately 20% [1, 49] for most adult brain tissues. Throughout the text the accent on superficial concentration has been dropped and it is denoted simply as *c*.

Upon careful examination of the concentration data, regions of distinctly different concentration dynamics are observed. These regions correlate with anatomical features of the brain and are observed in all experimental subjects. Concentration and $T_{1,0}$ thresholds were used to develop masks, voxels assigned to a specific feature, that segment the brain into volumes exhibiting different concentration dynamics. Since $T_{1,0}$ varies markedly between different types of tissue, it can be used to identify anatomical features, such as blood vessels and ventricles. Using $T_{1,0}$, the segmented volumes (described in “Results”) are shown to correlate with anatomical features.

### Finite-element model

Three-dimensional, finite-element models (FEMs) of transport in the mouse brain were developed based on the simplified transport equation (Eq. 2). Subject-specific models of the whole brain (skull and subarachnoid space excluded) segmented into five anatomical volumes, or subdomains, were built for four different mice. Effective diffusivities were varied for each anatomical subdomain to determine the optimal combination of parameters that give best agreement with the DCE-MRI concentration data.

To build the finite-element mesh, brain surfaces were extracted from the MRI data using MRIcroS [53]. Defects in the resulting surface were repaired using Meshlab [54]. The volume mesh of approximately 900,000 tetrahedral elements was generated with gmsh [55]. The anatomical masks were interpolated onto the mesh using an inverse-distance weighted (IDW) method to define anatomical subdomains within the whole-brain mesh. Time-series contrast concentrations were also interpolated onto the mesh for error calculations. The IDW method is essentially the application of a Gaussian filter, and therefore also had the effect of locally smoothing the data.

The simplified transport equation (Eq. 2) was solved by finite-element method within the complex geometry of the whole mouse brain. Effective diffusivity, $D_{eff}$, was assumed to be constant within each subdomain, but each subdomain might have a different $D_{eff}$ value. The contrast injection, which occurred over the first 20 min of the experiment, was modelled as a point source in space and a rectangular function in time. Although concentration data at the surface of the brain would have been the most accurate boundary condition, any contrast enhancement there was obscured by the strong local T2/T2* effect derived from the nearby skull, which decreases intensity and renders the data useless for a thickness of one to three voxels from the outer surface (see Additional file 2). (The SAS of the murine brain is extremely narrow, resulting in a minimal barrier between the brain and the skull.) A no-flux boundary condition was applied, given the skull is an impenetrable barrier, except for discrete locations like the opening for the spine. Although some contrast agent will leave the brain via CSF and glymphatic efflux routes, it is assumed this loss will be small over the short timescale of the DCE-MRI experiment.

The appropriateness of the no-flux boundary condition was tested by calculating the total amount of gadoteridol in the brain versus time using the concentrations calculated from DCE-MRI data. If a no-flux boundary condition accurately represents the physical situation, the total amount of Gad in the brain would increase during the injection, over the first 20 min, and then remain constant. The total amount of gadoteridol in the brain, calculated from DCE-MRI data, increases or plateaus over the full course of the experiment (up to 84 min) for three out of the four mice, consistent with no loss of contrast agent through the boundaries, or no flux. Observed increases in total gadoteridol over time are an artifact of T2/T2* interference with the T1-weighted contrast enhancement at high contrast concentrations, which exclude several voxels (on the order of 10,000 voxels) near the injection site containing relatively large amounts of gadoteridol from the calculation at early time points. As contrast disperses over time, the number of voxels effected by T2/T2* interference decreases, adding previously “hidden” gadoteridol to the total calculation.

Effective diffusivities were varied among the subdomains and the root mean square error (*rms*) between the data and the simulation mapped to determine the set of $D_{eff}$ resulting in the minimum error.

6 $$rms = \sqrt {\sum\nolimits_{c = 1}^{T} {\frac{{\left( {c_{data} - c_{simulation} } \right)^{2} }}{T}} }$$

The T2/T2* interference region near the injection site, the ventricles, and the blood vessels were excluded from the error calculation. Time points up to 52 min were used in the error calculation, later time points were excluded to minimize the error due to assumptions of no efflux routes and the no-flux boundary condition.

Equation 2 was approximately solved using FEniCS [56, 57], an open-source solver of partial differential equations by the finite-element method, using quadratic (Lagrangian) mesh elements and a preconditioned iterative solver for the linear system of equations. The time derivative was discretized using a backward difference (i.e., an implicit Euler method). The solution for each set of effective diffusivities required 2–3 h on an Amazon Web Server (AWS) c5 instance or a MacBook Pro with an i9 processor and 32 GB of RAM. Fifty to two-hundred combinations of $D_{eff}$ were simulated for each subject to generate error surfaces from which the values resulting in the minimum rms were determined (see Additional file 2). Post processing of simulations and DCE-MRI concentration data was carried out using Python [58], Paraview [59], and Excel. Every effort was made to use open-source software.

## Results

### Segmentation of transport regions

Careful evaluation of three-dimensional time-series DCE-MRI experimental data revealed several regions of enhancement with distinct concentration dynamics that aligned with the anatomy of the cerebral arterial vasculature and the cerebral ventricular system. In all subjects, contrast was observed to move from the injection site at the back of the brain along the major arteries of the ventral surface, then surrounding major branching arteries and from those preferential routes into the parenchyma. Based on these observations, the pre-contrast baseline images and DCE-MRI data were used to individually segment each brain into five volumes: (1) the cerebral ventricles, (2) the macroscopic cerebral arterial vasculature, (3) periarterial spaces surrounding the major surface arteries, (4) periarterial spaces surrounding major branching and penetrating arteries, and (5) the remainder of the brain parenchyma (Fig. 3A). Contrast was not observed surrounding major venous structures, in fact little tracer was observed in the dorsal half of the brain where venous structures are most prevalent in mice and rats. Lack of contrast near venous structures could be due to either: (1) contrast had not yet reached the major venous structures or (2) upon reaching major perivenous space the contrast was transported away so quickly that its concentration remained small. The five segmentations listed above were used to create subdomains within the finite-element model for which unique transport parameters were determined.

The MRI acquisition obtained isotropic 100 μm voxels, which through atlas registration were interpolated to 60 μm isotropic voxels, and provided sufficient resolution to segment the proximal arterial vasculature. The identifiable surface arteries include the communicating arteries of the Circle of Willis (CoW), basilar artery (Bas), and the anterior cerebral artery (ACA) (Fig. 3D). The identifiable branching arteries include the posterior cerebral artery (PCA), middle cerebral artery (MCA) and olfactory arteries (OAs) (Fig. 3D).

The periarterial spaces were identified based on their unique concentration dynamics and are named periarterial due to their location, surrounding the major arteries. High contrast concentrations are observed surrounding surface and branching arteries at early time points that describe the major periarterial space. Periarterial spaces surrounding the surface arteries (PAS_Surf_) and the branching arteries (PAS_Branch_) (Fig. 3B and C) were defined by high contrast concentration ([gadoteridol] > 0.15 mM at t = 20 min) and proximity (≤ 7 voxels for PAS_Surf_ and ≤ 3 voxels for PAS_Branch_) to major arteries (T_1,0_ < 1000–1400 (depending on subject) determined from the baseline (pre-contrast) MRI scans).

Preferential routes of tracer movement surrounding the surface arteries that transit the subarachnoid space have been established by several research groups [41, 60, 61]. The anatomical details of these preferential routes have been argued, however, independent of anatomy they have come to be referred to as periarterial space [41]. Harrison et al. further demonstrated that fluid flow in these preferential routes is both parallel and proximal to the arteries [62]. Pizzo et al. present fluorescent and DCE-MRI images of the preferential routes surrounding the major arteries on the ventral surface of the brain similar to the segmentation defined as surface periarterial space in this work [41]. Periarterial space in the segmented model does not necessarily denote anatomical periarterial space, but correlates well with the structure and width of the preferential transport routes characterized by Mestre et al. [18] and Tithof et al. [46] and identified by those groups as PVS surrounding arteries (see Additional file 2). In addition, the segmented periarterial volumes of the model serve the purpose of the preferential transport routes identified as periarterial space in the glymphatic model.

**Fig. 4:**
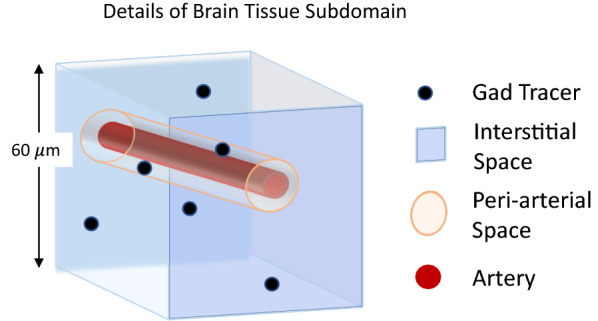
Simplified illustration of a DCE-MRI voxel, which measures 60 μm on a side, in the brain tissue subdomain (light blue in Fig. 3A). Each voxel in the brain tissue subdomain contains interstitial space and PVSs too small to resolve by MRI. Therefore, the transport parameter determined for this subdomain combines transport in the interstitial space and PVSs

At 60 µm resolution, smaller arteries and the microvasculature could not be individually resolved from the wider brain interstitium and were thus lumped into voxels of the brain tissue volume (Fig. 4). Therefore, $D_{eff}$ estimated for the brain tissue volume will lump, or combine, transport in interstitial and small PVS (periarterial and perivenous space). Contrast transport into the arteries and across the ventricular walls (between the CSF compartment within the ventricles and the surrounding brain tissue) was negligible and these regions were assigned extremely small transport parameters (0.000001% of the brain tissue region). Thus, based on the segmentation described above, the current model defines optimal parameters for three key regions in brain-wide transport: the PAS_Surf_, the PAS_Branch_, and the remaining brain tissue (BT), combining interstitial and small perivascular spaces.

### Transport parameters

The average $D_{eff}$ (and standard deviation) for each anatomical subdomain are reported in Table 1. The $D_{eff}$ for each subdomain is greater than $D_{app}$, indicating that transport is faster than attributable to diffusion alone throughout the brain. The estimated value for $D_{eff}$ is greatest for the regions surrounding the major arterial spaces (PAS_Surf_, PAS_Branch_) whose values are of similar magnitude. In fact, $D_{eff}$ in the periarterial regions is so much faster than $D_{app}$ (> 10,000× faster) that periarterial transport along major vessels can only be explained by convection. Dispersion in major periarterial spaces is predicted to increase the rate of transport over diffusion by a factor of as much as two [20]. Asgari et al. assumed porous periarterial space, while transport in periarterial spaces at the surface of the brain has been shown to agree with transport in open, non-porous channels [46]. In an open channel, predicted dispersion may be greater, but would still account for only a very small fraction of the observed enhancement. For transport in unhindered space, such as an open channel, $D_{eff}$ is more appropriately compared to the free diffusivity (*D*). $D_{eff}$ for the major periarterial spaces is > 1,000 X’s faster than *D*, also indicating convection.

**Table 1:** Quantitative analysis of brain transport parameters for each anatomical subdomain

Anatomical Subdomain	$D_{eff}$ (mm^2^/min)	Peclet number (open/porous)	Charac. length (mm)	Estimated velocity (mm/min)	Published transport parameter
Branching Periarterial Space (PAS_Branch_)	60 ± 20	4000/12,000	5	12 ± 4	$v_{PAS} = 1.2\,{\text{mm}}/{\text{min}}$ [18, 19]
Surface Periarterial Space (PAS_Surf_)	95 ± 40	6000/19,000	12	8 ± 3	$v_{PAS} = 1.2\,{\text{mm}}/{\text{min}}$ [18, 19]
Brain Tissue (BT): Interstitial space, PVS of microvessels	0.10 ± 0.04	6/18	0.5	0.2 ± 0.1	$D_{app} = 0.005\, {\text{mm}}^{2} /{\text{min}}$ [48, 49] $v_{IS} = 0.01\,{\text{mm}}/\min$ [32] $v_{PAS} =$ not known

Table reports: (1) Optimal effective diffusivity $D_{eff}$ . (2) *Peclet* number reflecting the ratio of convective to diffusive transport. “Open” uses the free diffusivity and represents transport in unhindered space such as surface periarterial space. “Porous” uses apparent diffusivity and represents transport through porous media such as interstitial space. (3) Average velocity estimated from $D_{eff}$ using a characteristic length for the anatomical subdomain. (4) Measured brain transport parameters (or parameters estimated using a model) from the literature for comparison. (Error is one standard deviation)

In the BT subdomain, which combines interstitial space and PVSs associated with smaller vasculature and the microcirculation, the enhancement of overall transport ($D_{eff}$) over $D_{app}$ is smaller (10–25×), but still supports the presence of convection within the bulk tissue. As discussed above, $D_{eff}$ of the BT volume represents a combination of diffusion, periarterial convection, periarterial dispersion, interstitial convection, and perivenous convection. For a small molecule like gadoteridol (550 Da), interstitial convection and diffusion are predicted to have similar rates [32]. Dispersion could also enhance transport within the smaller periarterial space, however, as discussed above, it is unlikely to have a contribution high enough to account for the full remaining enhancement of $D_{eff}$ within the BT subdomain over $D_{app}$. Therefore, much of the increase in $D_{eff}$ over $D_{app}$ observed in the BT volume is likely attributable to convection along the smaller penetrating PVS, in agreement with the observations of Cserr et al. and Rennels et al. [6, 7].

Effective diffusivity is a volume averaged value, while the perivascular space makes up only a small fraction of the brain volume, meaning the enhancement of overall transport compared to diffusion alone in the small PVS is likely much greater than that of the combined brain tissue region. Vasculature occupies about 3% of the total brain volume on average. If one assumes perivascular space is also about 3% of the brain volume, based on the PVS having a similar cross sectional area to the vessel it surrounds [18], then, after subtracting interstitial convection and periarterial dispersion, $D_{eff}$ attributable to PVS convection in the brain tissue is estimated at 3 mm^2^/min. This small PVS transport rate is 600×’s faster than $D_{app}$ and about 20×’s less than transport in the major periarterial space, suggesting perivascular flow continues into the brain parenchyma and may be slower than in the periarterial space surrounding major arteries. It is unknown whether perivascular space within the brain parenchyma is porous in nature, potentially filled with extracellular matrix [20, 25]. However, such rapid transport suggests open (not porous) PVS channels.

### Simulation comparison

**Fig. 5:**
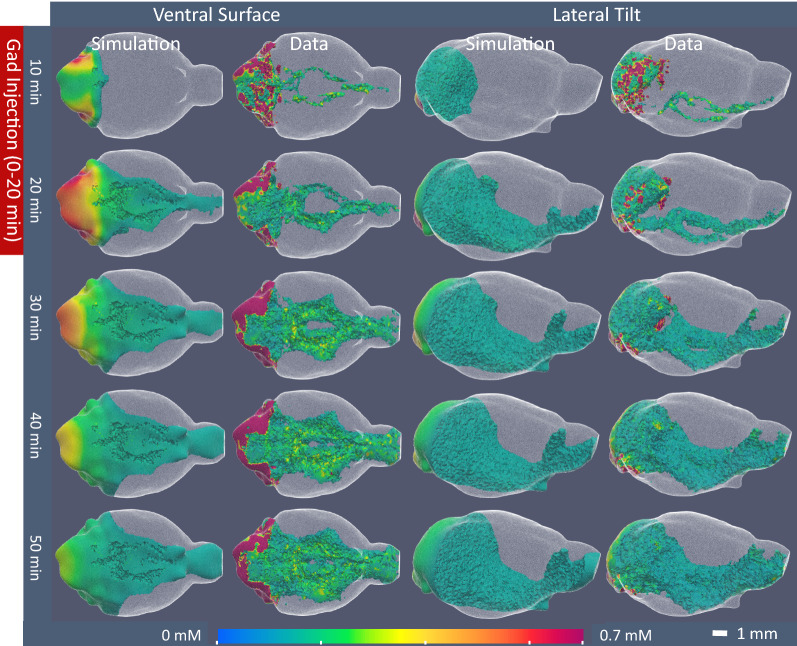
Comparison of simulation to experimental DCE-MRI gadoteridol concentration data. Images of contrast concentration versus time of simulation results for a representative mouse using segmentation as described above, and optimal transport parameters (brain tissue $D_{eff}$  = 0.1 mm^2^/min, PAS_Surf_ $D_{eff}$ = 95 mm^2^/min, PAS_Branch_ $D_{eff}$ = 60 mm^2^/min) compared to concentration calculated from DCE-MRI signal data. The 3D images show only the elements with concentration greater than 0.1 mM and are colored by concentration according to the color bar at the figure bottom. Images are shown for the ventral surface and from a lateral view with a slight downward tilt to display details around the Circle of Willis on the ventral surface and the branching arteries. In the data images, contrast is observed moving outwards from the injection site near the cisternal aqueduct, rapidly along the ventral surface of the brain following the communicating arteries of the Circle of Willis. Contrast then moves into the brain along major branching arteries, and from these preferential routes, penetrates the wider brain tissue. Contrast present in the ventricular system has been removed from the images for direct comparison to the simulation, which modelled only glymphatic transport (not ventricular transport). In the simulations, contrast movement follows the same progression, although it does not move as rapidly towards the anterior brain at early time points and remains higher in posterior regions than in the experimental data

Figure 5 shows simulated concentration contours compared to concentration calculated from DCE-MRI data for a representative mouse (see Additional file 1 for 3D animations and Additional file 2 for 2D slices). Contrast agent progression follows the same pattern from its posterior injection site as observed in other studies [34–40]: (1) contrast moves rapidly along the ventral surface of the brain following surface arteries, reaching the anterior brain in less than 10 min, (2) contrast follows branching arteries into the brain parenchyma, and (3) moves into the surrounding brain tissue from these major periarterial pathways. Given the simplifications applied in the present model, concentration simulations are visually well matched to the experimental data. In the simulation, however, contrast does not move as rapidly towards the anterior brain at early time points and the volume occupied by contrast concentration > 0.1 mM remains greater in the posterior brain compared to the observed experimental distribution. This discrepancy is an outcome of modelling convective transport, which has a first-order relationship with concentration gradient (Eq. 1), using a diffusive model, which is second-order with respect to concentration gradient (Eq. 2). A second-order model, such as that used in the simulation, will yield higher concentration near the contrast source (the posterior brain) and decrease more rapidly moving anteriorly, while in a first-order model, such as that of convection in the full transport equation, concentration will follow a linear decline. The observed first-order relationship between contrast concentration data and rostro-caudal distance is additional evidence in support of periarterial convection.

A more refined inspection of the simulation versus the experimental data (Fig. 5 and Additional file 2) shows that at later time points the experimental data exhibit heterogeneity of contrast concentration (e.g., distinct areas of high and low concentrations), while in the simulation the contrast concentrations decline smoothly through the tissue. This smooth dispersal is the expected outcome of a diffusive model (Eq. 2). Therefore, such discrepancies are expected given the model chosen and the anatomical simplifications made in this work. The heterogeneity of the measured contrast concentration at later time points suggests that the brain tissue is more heterogeneous than modelled, as are the transport mechanisms underlying this anatomical heterogeneity. Certainly, the local agreement between the simulations and the experimental data can be improved by including greater anatomical detail. However, the addition of these details adds complexity and requires more adjustable parameters that dilute the usefulness of the quantified parameters, add new potential sources of error and require extremely large computational resources. Although the model does not perfectly simulate the experimental data, the parameters determined from the model well describe macroscopic transport mechanisms throughout the brain.

## Discussion

DCE-MRI experiments have long reported transport in the brain that appears to be faster than diffusion alone, but this transport rate has not been quantified to a physically relevant value that can be compared to known diffusive rates of contrast agents. The effective diffusivities ($D_{eff}$) estimated from our analysis are orders of magnitude larger than diffusivity of the contrast agent through brain tissue ($D_{app}$), and thus indicate that convection is a significant contributor to transport throughout the brain. In this *Discussion* section, the $D_{eff}$ results are further analyzed using concepts from transport phenomena and the results of that analysis compared to values reported in literature. Further, experimental factors impacting transport and sources of error from the analysis and the experiments are explored.

### Mechanisms of transport

Péclet number (*Pe*) is a dimensionless group that assesses the relative importance of convective to diffusive transport rates:

7 $$Pe = \frac{rate \, of \, convection}{{rate \, of \, diffusion}} = \frac{chateristic \, diffusion \, time}{{characteristic \, convection \, time}} = \frac{{L^{2} /D}}{L/v} = \frac{Lv}{D} = \frac{{D_{eff} - D_{app} - D_{disp} }}{{D_{app} }}$$

If transport is predominantly by diffusion (i.e., by random molecular motion in a stagnant fluid), then *Pe* ≪ 1, and if transport is predominantly by convection (i.e., by bulk fluid motion), *Pe* ≫ 1. Because it is uncertain whether perivascular space is open or porous, *Pe* is calculated using both *D* and $D_{app}$ respectively. Table 1 reports *Pe* for each anatomical region using *D* = 0.016 mm^2^/min, $D_{app}$ = 0.005 mm^2^/min (Eq. 3) and $D_{disp} = D_{app}$ [20]. In both major periarterial spaces (PAS_Surf_ and PAS_Branch_), which are believed to be open, *Pe* exceeds 1000 and convection (or bulk flow) is the dominant mechanism of transport. The brain tissue (BT), which consists of a combination of interstitial tissue and PVSs associated with small blood vessels, exhibits an intermediate *Pe* = 18, where convection and diffusion are both relevant, but convection remains the larger mechanism of transport for the contrast agent. From the Péclet analysis, we conclude convection is present within and significant to transport within the wider brain tissue volume, however the contribution of each of its physiological components (the bulk interstitium versus microvascular PVS) is not determinable.

It is also important to note the transport characteristics of the contrast agent used in this DCE-MRI study. The contrast agent was chosen for its lack of interaction with cells and its absence of known carriers or transporters in the CNS; it is biologically inactive. Therefore, its transport is not slowed by biological interactions and is indicative of the pure transport mechanisms of diffusion, dispersion, and convection. Biologically active molecules will be slowed by cellular and transporter interactions, with their resulting rate being molecule specific. The transport rates observed in the DCE-MRI data and analyzed here should be considered the upper limit of transport for molecules around the size of gadoteridol (559 Da). Gadoteridol is also a smaller molecule compared to biological macromolecules, including peptides and proteins of interest in neurodegeneration, such as amyloid β (4.5 kDa). These larger molecules have smaller $D_{app}$, and therefore larger *Pe*, meaning the convection observed here will have an even greater effect on the overall transport of such macromolecules.

### Average velocity

Using the definition of Péclet number (Eq. 7) and a characteristic length for transport in each region, $D_{eff}$ can be used to estimate average fluid velocity (*v*), which is reported for each region in Table 1. Periarterial convection is estimated to have an average velocity of $v_{PAS}$  = 8 mm/min in the PAS_Surf_ and $v_{PAS}$ = 12 mm/min in the PAS_Branch_. The choice of characteristic length has a significant impact on the velocity calculated in this way. Using the convention of computing characteristic transport times, characteristic lengths in the primary direction of transport for each anatomical region were estimated. The characteristic lengths reported in Table 1 for the PAS_Surf_ and PAS_Branch_ were chosen based on the length dimension of the brain in each direction (caudal-rostral for PAS_Surf_ and ventral-dorsal for PAS_Branch_). As the vasculature followed by the PVS takes a tortuous, branching path and is connected from the major arteries down to the capillaries, the appropriate characteristic length for the periarterial regions may be longer than estimated, which would result in lower velocities. Perivascular fluid velocity at specific locations along the MCA (and its immediate branches) was measured to be 1.2 mm/min by both Mestre et al. and Bedussi et al. [18, 19]. The measured velocities are slower than the velocities estimated in our analysis, but similar in order of magnitude. The difference in velocity values may derive from: (1) differences in transport between small chemical tracers and particles, (2) calculation of an average velocity over a system of periarterial spaces versus measurement of particle velocity at a specific location, and/or (3) sources of error in both the analysis and the experiments.

The BT volume represents a combination of interstitial transport and perivascular transport surrounding smaller blood vessels (illustrated in Figs. 1 & 3B). Therefore, literature values are investigated for each and compared to the estimated average velocity of $v_{BT}$ = 0.1–0.3 mm/min (Table 1). In previous work, Ray et al. estimated interstitial flow in the brain (which is expected to be the lowest velocity for all anatomical regions considered here) on the order of $v_{IS}$ = 0.01 mm/min [32]. Measurements of interstitial flow through tissues outside the brain (in the periphery), which likely represent an upper limit on brain interstitial transport, report $v_{IS}$ = 0.006–0.12 mm/min [63]. Both estimates of interstitial velocity are lower by an order of magnitude than the average velocity estimated from the $D_{eff}$ in the lumped BT volume, indicating the likelihood of a significant contribution from PVS flow.

Another way to think about velocity in the brain tissue region is as the velocity of a transport ‘front’. The smaller penetrating arteries and microvessels branch out in many directions, so there may be no clear direction of flow on the scale of the BT volume either in the penetrating periarterial and perivenous spaces or for the interstitial flow that runs from periarterial to perivenous space. Instead, contrast progresses more rapidly than by diffusion alone, but along a tortuous convective path. Therefore, the velocity calculated in Table 1, $v_{BT}$ = 0.1–0.3 mm/min, may be better described as the velocity of a ‘front’ progressing deeper into the brain, as opposed to the velocity of the actual interstitial or perivascular flow. Plog et al. measured front velocities on the order of 0.1–1 mm/min in mice in the space between large vessels (analogous to the BT volume in this work) [64]. This front velocity, or an enhancement factor of $D_{eff} /D_{app}$, may be a better representation of transport for the combined effects of periarterial convection, perivenous convection, interstitial convection, dispersion, and diffusion in the brain tissue.

The results presented above for both the periarterial spaces of major branching arteries (PAS_Branch_) and the periarterial space of smaller penetrating arteries (included in the BT volume) support continuation of periarterial flow into the brain along penetrating arteries. Mestre et al. [18] and Bedussi et al. [19] reported that 1-μm microspheres tracking fluid movement in the periarterial space surrounding the MCA were excluded from smaller branches of that artery penetrating into the brain. This observation raised the question of whether periarterial flow continues into the brain, and if so, whether the penetrating periarterial spaces are filled with protein networks that behave like a porous media and impede flow. In the model, the PAS_Branch_ is comprised of PVS of the MCA, which resides primarily on the surface of the brain, but also the posterior cerebral artery (PCA) and the OAs, which penetrate into the brain tissue. Convection was apparent along these large arteries of the PAS_Branch_ at a magnitude that suggests open channel flow, not flow through porous media. Convection is also supported for the space of microvessels. Although the actual flow velocity in the perivascular space of smaller arteries and veins cannot be determined from this analysis performed at the scale of the whole brain, the results are best explained by significant perivascular flow surrounding penetrating vasculature. Therefore, the results reported here, when combined with current understandings in the literature, support open-channel periarterial flow along large penetrating arteries and perivascular flow along smaller cerebral blood vessels that may be open-channel or porous media flow.

### Effect of experimental factors on transport rate

Although DCE-MRI is widely used for assessing solute transport in the brain, factors affecting glymphatic transport continue to be investigated and experimental techniques are still being standardized. Experimental parameters that may affect the glymphatic transport rates the DCE-MRI experiments are designed to measure include: contrast infusion procedures, body posture, and anesthesia. First, injection of contrast is accomplished through a pipette surgically inserted into the cisterna magna (CM), a widening in the subarachnoid space (SAS) surrounding the brain that is filled with CSF, at a controlled rate over several minutes. Lundgaard et al. reported a decrease in glymphatic function with CM puncture under conditions where CSF was allowed to drain out of the skull [65]. The decrease in glymphatic function was believed to be a result of the associated loss of intracranial pressure, not the puncture itself. In the DCE-MRI experiments, the needle/pipette removal coincided with sealing of the dura with cyanoacrylate glue. Thus, while needle removal opening the CM does impair glymphatic function, the sealing approach applied here maintains normal levels of glymphatic function like those observed where the needle is left in place.

Second, body posture has been shown to affect transport rates in the murine brain, with the highest rates being observed in the lateral position and higher rates in the supine than prone position [66]. Rodents sleep naturally in groups, which can lead to a wide variety of body postures [33]. Foundational glymphatic experiments (prior to DCE-MRI) were performed in the prone position [6, 7, 10, 34] and DCE-MRI images reported in literature are acquired in either the prone or supine position [17, 33, 34, 67, 68], or the position is not reported [39, 69]. Based on contrast retention rates and loss rates calculated using a two-compartment model, brain transport in the prone position is 40% slower than in the supine position and about half the rate in the lateral position [66]. The DCE-MRI data used in this analysis was collected from mice in the prone position, therefore transport is likely to be slower than for data collected in the supine position.

Finally, anesthesia type is known to effect brain transport rate [35–39, 70]. Most DCE-MRI experiments in the literature utilize isoflurane [33], which is an inhalable anesthesia standardly used in MRI, while a few use ketamine-xylazine (K-X), which was standard in many of the foundational experiments (prior to DCE MRI) studying the glymphatic system. In particular, Xie et al. showed K-X to mimic natural sleep with respect to glymphatic activity [10]. Stanton et al. recently confirmed that contrast transport pathways differ between isoflurane and K-X anesthetic regimes [71]. Specifically, parenchymal penetration is poorer using isoflurane due to rapid efflux of contrast agent into the spinal canal and along cranial nerve sheaths. Under K-X anesthesia, contrast is distributed into the brain parenchyma, transported to the olfactory bulb (a major efflux route), or transported down the spinal canal [71]. In the experiments used in this analysis mice were under ketamine-xylazine (K-X) anesthesia. As such, transport rates estimated here are expected to be faster than for data collected using isoflurane anesthesia.

### Sources of error

#### Segmentation

To estimate transport parameters for regions of the brain demonstrating different transport dynamics, the whole-brain model was segmented into volumes defined by either anatomical features determined from pre-contrast images and/or contrast concentration. DCE-MRI measures the average signal for the volume contained in each voxel. Therefore, at the boundaries between segmented volumes a voxel may contain more than one anatomical feature and the signal measured is an average of the signals resulting from each feature. Since the voxel size, or resolution, of 100 μm is significant with respect to anatomical features, “mixing” of different tissues at the boundaries of the segmented volumes is unavoidable. For example, there will be some fraction of brain tissue included in the periarterial volumes and vice versa. This “mixing” at the boundaries can be a source of error in estimating separate transport parameters, because there is not a clean division between features and concentration calculated from signal near boundaries includes contributions from each feature.

Valnes et al. experienced significant complications due to ‘voxel averaging’ between tissues at the interface between the SAS CSF and the brain in their analysis of human brain transport using finite element modelling [43]. Concentration at the CSF/brain interface was critical to their modelling as contrast was injected intrathecally and therefore entered the brain by passing from the CSF to the brain tissue. For the data analyzed here, contrast was infused directly into the brain volume resulting in less dependency between the analysis results and accurate contrast concentration at the surface of the brain. Errors in the data exist at the surface of the brain for the murine experiments analyzed here, but their source is not a segmentation error. In the murine brain, the surface signal is obscured by interference from local T2/T2* effects from the skull, which has closer proximity to the brain than in humans (see “Methods” and Additional file 2). The DCE-MRI data show extremely low signal at the surface of the brain to a thickness of approximately one to three voxels for the entire time course, verifying this interference. If contrast were present in the CSF surrounding the brain, a concentration gradient would be expected from the surface into the brain tissue. However, analysis of concentration gradients near the surface (see Additional file 2) shows no evidence of contrast agent in the SAS CSF. The effected surface voxels, which are included in the brain tissue region of the model, add to error between simulations and the data. However, this error is relatively consistent across different effective diffusivities, and is therefore unlikely to impact optimal effective diffusivity results, determined from minimum error.

With respect to other interfaces between transport regions, the BT volume is 99.9+ % of the total volume (by number of vertices), therefore, any PAS concentration dynamics incorporated into the BT volume at its boundaries are likely to have very little impact on the estimated BT $D_{eff}$. The estimated $D_{eff}$ for the PAS_Surf_ and PAS_Branch_ volumes are significantly greater than the BT $D_{eff}$ (> 600×s), therefore, any BT concentration dynamics incorporated into the PAS volumes at the boundaries are also likely to have little impact. In conclusion, although exact segmentation of features is limited by the resolution of the DCE-MRI measurements, such limitations are not likely to impact the transport parameter estimates which are significantly different between regions sharing boundaries.

#### Transport model

Transport in the brain is part of a complex, dynamical interaction involving the vasculature (arteries, veins, and microvasculature), the CSF circulation, brain tissues, fluid/solute exchange, and the cranio/spinal connection. Transport may be influenced by pulsation [9, 18], cycles [27], and electrical waves [72] with short time constants relative to the DCE-MRI data being analyzed. The goal of the present study is to estimate fundamental transport parameters that describe general transport over the macroscopic scale of the whole brain with the intention of better understanding the prevalence and magnitude of convection or dispersion in molecular transport across the entire brain. The goal is not to build a mechanistic model including refined details of brain transport and anatomy, but to improve on current analysis of DCE-MRI data by applying the rigors of transport phenomena to determine physically relevant transport parameters. In order to analyze the data without bias to transport mechanism, the transport equation was simplified condensing all mechanisms into a single term; and in order to develop a computationally feasible model, anatomical features of the brain were substantially simplified. Although including additional anatomical subdomains (with unique $D_{eff}$) would have led to improved agreement between simulations and data, it would also have resulted in additional adjustable parameters that diluted the usefulness of the results. The simplified model resulted in parameter estimates that are an improvement over current analysis methods and provide understanding about transport routes and mechanisms that will help pave the way to more rigorous transport models in the future.

The simplified transport equation also forces the mathematical structure of diffusive transport. Careful comparisons of data and simulations show that the relationship between concentration and distance, particularly in the perivascular space, better follows the mathematical form of convection. The model could be greatly improved by specifically including all mechanisms of transport as shown in Eq. 1. However, the information required to build a convective model (e.g., pressure gradients driving physiological flow) at the scale of the whole brain is currently unknown. Alternatively, a convective field could be measured experimentally, possibly by Intravoxel Incoherent Motion (IVIM) MRI [73]. The velocity field could be applied to the finite-element mesh directly and the transport model could be used to answer more subtle questions about brain transport, i.e., understanding efflux routes or modelling disease states.

The transport model assumed a no-flux boundary along the brain surface and did not include efflux routes for the contrast agent to exit the brain volume. Although the skull presents an impenetrable (no-flux) barrier for much of the surface, it also has specific routes of efflux, namely the spinal cord, the cranial and olfactory nerves, and meningeal lymph vessels. Inspection of unmasked DCE-MRI data shows the presence of contrast along the spinal cord, especially at early time points. It is difficult to quantify the amount of contrast agent lost to the spinal cord, however, signal in the spinal cord is much less than signal observed in the parenchyma surrounding the injection site or surrounding the surface and penetrating arteries, and also less than the contrast concentration in the parenchyma surrounding these periarterial spaces.

In the glymphatic model, fluid and molecules hypothetically exit the brain tissue through major perivenous space, ultimately emptying to extracranial lymphatic vessels outside the blood–brain barrier [8]. As discussed in the “Results” section, no contrast was observed surrounding major venous routes that reside in the dorsal half of the murine brain, therefore no data was available to inform transport along these specific pathways. In addition, glymphatic efflux routes are not well understood and are therefore a place of continued investigation [22, 25].

If efflux or loss through the no-flux boundary was significant to transport over the time scale of the experiments, the total amount of tracer in the brain would be expected to decrease steadily after the injection was complete. However, calculations made from DCE-MRI data show no decrease in total gadoteridol amount over the time points used in the analysis (0–54 min) for three of the four subjects, which supports that efflux may not be a significant contributor to transport over the time scale investigated. As more clarity is gained about mechanisms of efflux, distributed efflux or specific routes may be added to the model to improve its representation of the physical situation and allow analysis of longer time-series data.

#### DCE-MRI experiments

Even though it is a powerful experimental tool, MRI is an inherently noisy technique with a low signal-to-noise ratio. In live biological subjects, this noise is exacerbated by small movements in the tissue, such as blood pumping through vessels, or movement of the entire subject. MRI data is often filtered to reduce noise and smooth the data, but at the cost of averaging the data over regions considered large relative to anatomical details. In order to investigate the interaction of transport with specific anatomical features, the decision was made to leave the data unfiltered. (Some minimal Gaussian filtering occurred as a result of interpolating the MRI data onto the finite-element mesh.) A consequence of using the unfiltered data is greater variability between points in space and time, leading to greater error between the simulation and the data. However, all parameter combinations experience this same noise, and the transport parameters giving the minimum difference between simulation and data, which is relative, should not be greatly affected.

The DCE-MRI data used in this analysis exhibited T2/T2* interference at high contrast concentration, sacrificing useful data near the injection site. The DCE-MRI experimental parameters (i.e., magnetic field and pulse sequence) were optimized for measuring low concentrations of gadoteridol in order to follow it deep into the tissue and possibly elucidate efflux routes. In particular, T2/T2* interference obscured the rapid transport path from the injection site to the tissue surrounding the ventral surface arteries, adding uncertainty to the PAS_surf_ geometry used in the model. T2/T2* interference was more likely to increase variability than to result in higher or lower values for $D_{eff}$.

It has been questioned whether the contrast infusion, 0.5 μl/min for the first 20 min of the experiments used here, might induce convection [28]. Xue et al. established infusion rates of 1–2 μl/min in mice to be safe and associated with only minor transient changes in intracranial pressure (ICP) [74]. Raghunandan et al. directly addressed this question by comparing a tracer study with traditional infusion techniques, where tracer solution is slowly injected into the CSF, to a study where CSF was removed from the cranium at the same rate the tracer solution was injected, for no net addition of fluid [75]. The experimental results, where periarterial velocity in mice was measured using the same methods as Mestre et al. [18], were statistically identical for the two cases. The infusion rate used by Raghunandan et al. was four times higher than the infusion rate for the DCE-MRI experiments reported here. In addition, Bedussi et al. [19] observed no increase in intracranial pressure (ICP) (CSF pressure remained within the range of pre-infusion ICP) at 70% of the infusion rate used in the experiments reported here. Therefore, published experimental results support that, at the infusion rate used for the DCE-MRI experiments analyzed here, convection is not an artifact of tracer infusion.

The model developed in this work could also aid in assessing this concern, but analysis of the current data is limited by T2/T2* interference in the important region near the injection site and few data sets during the infusion. Even with those limitations, a simulation fitting different $D_{eff}$ parameter sets during and after infusion showed potential differences, but still indicated significant convection in the PVSs surrounding major arteries and transport that is faster than diffusion alone in the brain tissue region post infusion.

### System efficiency

Finally, we analyze the transport parameter values as an integrated system. Constructal Theory, which has been used to analyze the efficiency of biological systems [76, 77], states natural systems optimize over time and optimize around the constraints of the space allocated to the ‘function of the system’, the functional space. In an optimized natural system, transport across the functional space will be the rate limiting step, i.e., have the longest characteristic transport time (τ) in the system, and the remaining ‘flow system’, will optimize around the functional space. For the glymphatic system, the functional space is the interstitial space, where nutrients are delivered to the cells and waste products are carried away, while the PVS makes up the flow system. Using $D_{eff}$ and characteristic lengths discussed above, τ(interstitial space) is about 3 min for small molecules (100 Da) and 13 min for large molecules (1000 Da), while τ(major periarterial space) = 1–3 min and τ(minor periarterial space) = 2–5 min. For transport of small molecules, τ is similar for each ‘step’, indicating the whole system is optimized. For transport of large molecules, the interstitial space is rate limiting as predicted, likely limited by the inherent slow diffusivity of large molecules and constrained by the maximum flow velocity tolerated by brain cells, and the flow system is faster.

## Conclusions

Quantitative analysis of DCE-MRI data for transport of the small molecule gadoteridol (550 Da) across the whole mouse brain using a simplified finite-element transport model, indicates convection is present throughout the brain. Convection is estimated to be dominant in the periarterial space of major surface and branching arteries, where *Pe* > 1000. Importantly, convection is estimated to continue into the brain tissue, demonstrating convection is more than a surface phenomenon. Perivascular convection for smaller penetrating vessels could not be separated from interstitial convection as the DCE-MRI data lack the resolution to make this distinction. However, comparison to estimated and upper bound values for interstitial flow and periarterial dispersion suggests fluid flow in the perivascular spaces dispersed throughout the brain tissue. This convection within the parenchyma is estimated to be relevant for small molecules like gadoteridol (550 Da), and significant to overall transport for larger molecules implicated in neurodegenerative disease.

The whole-brain transport model described in this work represents an improvement over previous DCE-MRI analysis methods in its quantification of fundamental transport parameters (instead of transport rates with arbitrary units) that can be directly compared to known diffusion rates. The model in its current form is simple, and not mechanistic, but is structured for continuous improvement as new information comes to light and may also be appropriately connected with small-scale models, such as for interstitial transport [32], to develop a multi-scale transport model. The transport analysis described here could be improved with experimental data investigating molecules of different sizes, i.e. 500 Da and 10,000 Da. Diffusion and dispersion are dependent on molecular size, while convection is relatively independent of molecular size. Therefore, comparison of transport data and parameters from contrast agents of significantly different molecular size would provide further evidence towards convective versus diffusive or dispersive transport. Additionally, IVIM MRI data, quantifying average fluid velocities throughout the brain, would enable improved mechanistic complexity in the transport model.

## Supplementary Information

**Additional file 1:** 3D animations of concentration data and representative simulation.

**Additional file 2:** Supplemental Material consisting of: 2D slices of concentration data and representative simulation, pre-contrast images and tissue properties, determination of contrast-agent relaxivity, example error contours for determining optimal transport parameters, periarterial-width sensitivity analysis and comparison to literature, and example FEniCS/python code for transport model and simulation.

## Data Availability

The datasets and computer code supporting the conclusions of this article are available from the corresponding author on reasonable request. An addition, an example code is provided in the Additional file.

## Abbreviations

ACA: Anterior cerebral artery
Bas: Basilar artery
BT: Brain tissue (combined interstitial space and perivascular space of smaller arteries)
CM: Cisterna magna
CoW: Circle of Willis
CSF: Cerebrospinal fluid
DCE-MRI: Dynamic contrast-enhanced MRI
*D*: Free diffusivity
*D*_*app*_: Apparent diffusivity
*D*_*disp*_: Dispersion coefficient
*D*_*eff*_: Effective diffusivity
FEM: Finite-element model
IVIM MRI: Intravoxel incoherent motion MRI
K-X: Ketamine-xylazine (anesthesia)
MCA: Middle cerebral artery
MRI: Magnetic resonance imaging
OA: Olfactory artery
PAS: Surface periarterial space
PCA: Posterior cerebral artery
*Pe*: Péclet number
PVS: Perivascular space
*rms*: Root mean square error
SAS: Subarachnoid space
$v_{IS}$: Interstitial superficial velocity
$v_{PAS}$: Periarterial space velocity
