# Supplementary figures and images for: Quantitative analysis of macroscopic solute transport in the murine brain
Source: Fluids Barriers CNS. 2021 Dec 7;18:55. doi: 10.1186/s12987-021-00290-z (PMC8650464; doi:10.1186/s12987-021-00290-z)

## Slide 1
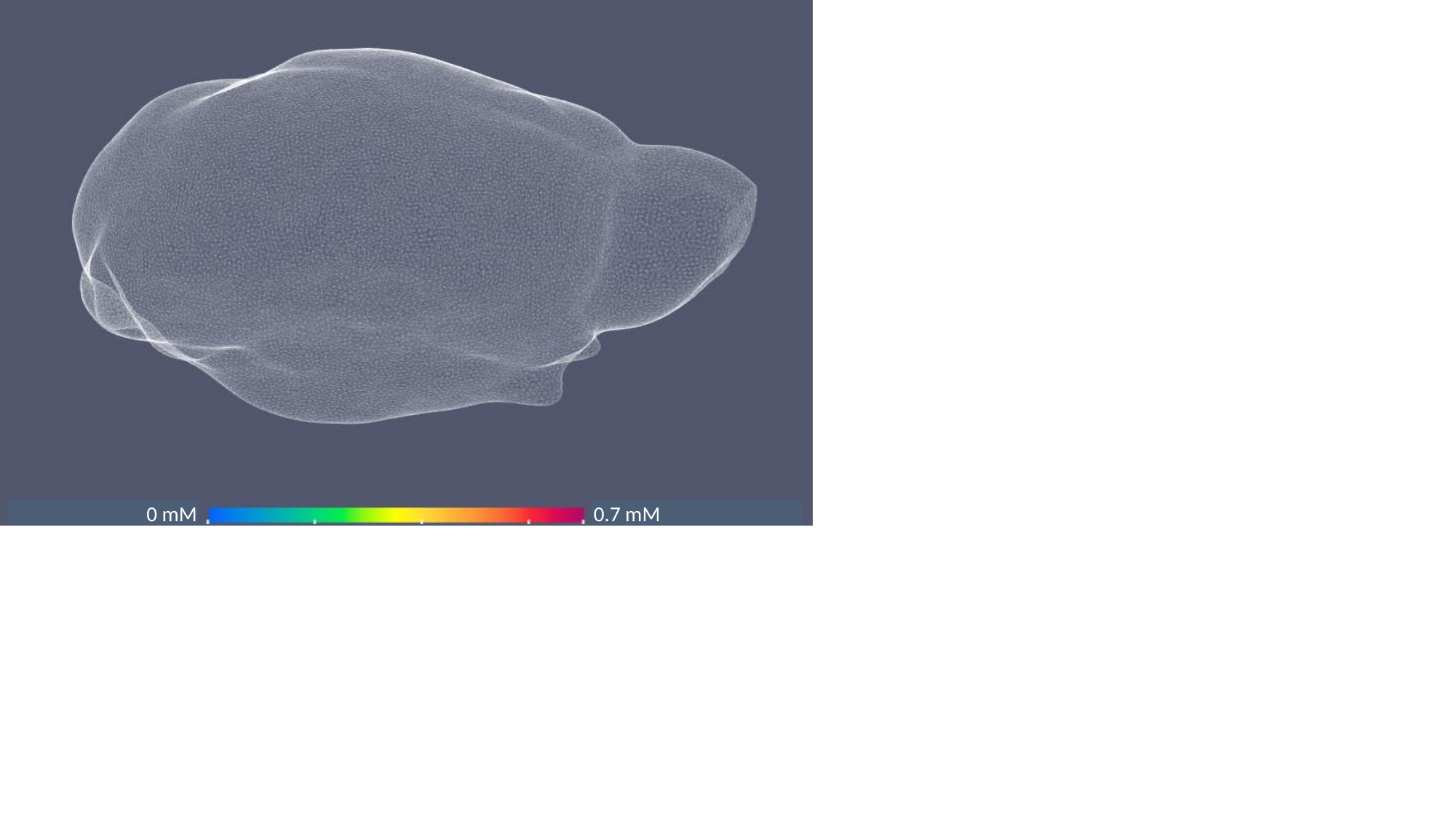

0 mM 0.7 mM

Supplement: Supplementary file 1 — Additional file 1: 3D animations of concentration data and representative simulation. [file 12987_2021_290_MOESM1_ESM.zip › FBCNS_FigS1.pptx]

## Slide 1
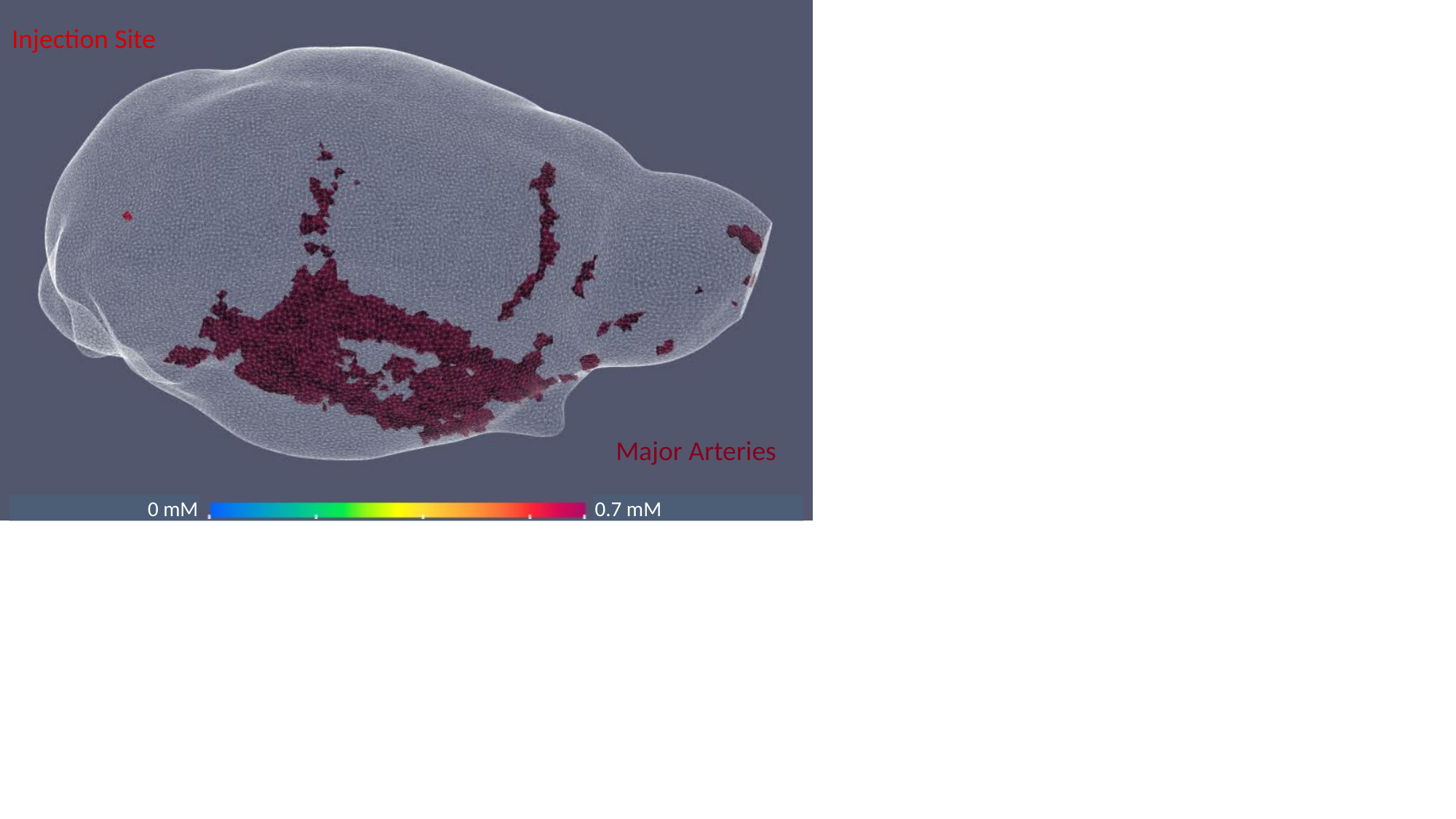

Injection Site
Major Arteries
 0 mM 0.7 mM

Supplement: Supplementary file 1 — Additional file 1: 3D animations of concentration data and representative simulation. [file 12987_2021_290_MOESM1_ESM.zip › FBCNS_FigS3.pptx]
