# Supplementary material for: Quantitative analysis of macroscopic solute transport in the murine brain
Source: Fluids Barriers CNS. 2021 Dec 7;18:55. doi: 10.1186/s12987-021-00290-z (PMC8650464; doi:10.1186/s12987-021-00290-z)
Supplement: Supplementary file 2 — Additional file 2: Supplemental Material consisting of: 2D slices of concentration data and representative simulation, pre-contrast images and tissue properties, determination of contrast-agent relaxivity, example error contours for determining optimal transport parameters, periarterial-width sensitivity analysis and comparison to literature, and example FEniCS/python code for transport model and simulation. [file 12987_2021_290_MOESM2_ESM.docx]

**Supplemental Material for “Quantitative Analysis of Macroscopic Solute Transport in the Murine Brain”**

1. **Animations and Slices of Contrast Concentration**
   1. DCE MRI Data

A 3-dimensional animation of the contrast concentration calculated from DCE-MRI signal is given in Figure S1.

**Figure S1**. Animation of Contrast Concentration calculated from DCE-MRI signal. The 3D images show only the elements with concentration greater than 0.1 mM and are colored by concentration according to the color bar at the figure bottom. Images are shown from a lateral view with a slight downward tilt to display details around the Circle of Willis on the ventral surface and the branching arteries. Contrast is observed moving outwards from the injection site near the cisterna magna, rapidly along the ventral surface of the brain following the communicating arteries of the Circle of Willis. Contrast then moves into the brain along major branching arteries, and from these preferential routes, penetrates the wider brain tissue. Contrast present in the ventricular system has been removed from the images for direct comparison to the simulation, which modelled only glymphatic transport (not ventricular transport).

Two-dimensional “slices” with relevant anatomy, such as major surface and branching arteries, for a representative mouse are shown in Figure S2. Upon careful examination of the concentration data, regions of distinctly different concentration dynamics are observed. These regions correlate with anatomical features of the brain and are observed in all experimental subjects. From its injection site at the posterior (back) of the brain, tracer moves rapidly anterior (forward) along the ventral (bottom) surface of the brain following the major arterial system made up of the basilar artery (Bas), the Circle of Willis (CoW), and the anterior cerebral artery (ACA). The preferential route along the surface arteries is best illustrated in the cross section of the Circle of Willis, where the tracer path clearly splits into two branches, following the branched arteries. Along this route, contrast moves from the posterior to the anterior of the brain (approximately 12 mm) by the first time point (10 min)—a rate of 1.2 mm/min or faster.

**Figure S2**. Contrast agent (Gad) concentration contours calculated from DCE MRI Data. Scale is c=0 (blue) to c=0.5 mmol (red). Concentrations exceeding 0.5 mmol are dark red (top of the color bar). The first column shows the center sagittal slice. The next three columns show coronal slices at different locations in the brain associated with major arteries and preferential transport routes. From its injection point in the CSF aqueduct at the posterior of the brain, contrast moves along preferential routes first along the system of arteries on the ventral surface of the brain, then along major branching arteries in the superior direction. From these preferential routes, contrast moves outward into the brain. Coronal slices illustrate this behavior at major arterial features. T2/T2* Interference: Occurs at high contrast agent concentrations ([Gad]>1.7 mmol) obscuring the T1-weighted signal used to calculate contrast concentration. Ventricular Accumulation: In addition to moving into the SAS, contrast also moves into the fourth ventricle and spreads by dispersion and diffusion throughout the ventricular system over the course of the experiment. Since contrast agent transport across the ventricular walls and into the brain tissue is negligible, transport through the ventricular system is effectively independent of transport into and through the brain tissue.

As contrast reaches the length of the brain along this ventral route, it also moves superior (upwards) through the brain at an equally rapid pace. High contrast concentration is observed around large arteries that branch from the surface arteries in the superior direction—the posterior and middle cerebral arteries (PCA and MCA), and the olfactory arteries (OA). Contrast penetrates the brain tissue from these primary routes. The above observations are consistent with previously published DCE-MRI experiments (1-4). Contrast is also observed to move outwards directly from the injection site at the aqueduct between the fourth ventricle and the cisterna magna into the brain tissue.

Contrast agent transport across blood vessel walls is negligible due to the blood-brain barrier, therefore, the arteries are obvious from their absence of tracer, amid high concentrations in the brain tissue. (Seen most clearly at intermediate and later time points in the olfactory bulb and on the ventral surface, Figure S2.)

- 1. Representative Simulation

A 3-dimensional animation of the contrast concentration simulated using the set of optimal effective diffusivities is given in Figure S3.

**Figure S3**. Animation of Contrast Concentration simulation versus time using optimal transport parameters (brain tissue $D_{eff}$ = 0.1 mm^2^/min, PAS_Surf_ $D_{eff}$ = 95 mm^2^/min, PAS_Branch_ $D_{eff}$ = 60 mm^2^/min) every minute for 50 minutes following the initiation of the 20-minute infusion. The 3D images show only the elements with concentration greater than 0.1 mM and are colored by concentration according to the color bar at the figure bottom. The arterial subdomain extracted from the concentration data is shown in deep red for reference. Contrast penetration with time is similar to the concentration data. However, in the simulation, contrast moves anterior more slowly along the surface arteries and superior more quickly along the branching arteries. This difference is likely due to the use of a diffusive model for convection dominated transport along the major arteries.

Figure S4 shows simulated concentration contours compared to DCE-MRI data for a representative mouse. Simulations visually match the data well at early time points and deviate more at later time points. At later time points the data exhibits continued heterogeneity of concentration (distinct areas of high and low), while in the simulation, the tracer smoothly disperses through the tissue. This smooth dispersal is the expected outcome of a diffusive model (Eqn. 2). Therefore, given the model chosen and the anatomical simplifications made in this work, a different outcome is not possible. The heterogeneity of the tracer concentration data at later time points tells us the tissue is more heterogeneous than modelled, as is the transport underlying the anatomical differences.

**Figure S4**. Comparison of contrast concentration simulated for the optimal set of effective diffusivities for a representative mouse ($D_{eff, BT}$ = 0.08 mm^2^/min, $D_{eff, SPAS}$ = 130 mm^2^/min, $D_{eff, BPAS}$ = 80 mm^2^/min). Simulations visually match the data well at early time points. At later time points, the data exhibits continued heterogeneity of concentration (distinct areas of high and low), while, in the simulation, the contrast smoothly disperses through the tissue. This smooth dispersal is the expected outcome of a diffusive model (Eqn. 2). The intention of the model was not to exactly match the data, but to calculate transport parameters that are useful estimates for each broad region of fluid movement in the brain.

Certainly, local agreement between the simulations and data can be improved by including more anatomical details. However, the addition of these details adds complexity and requires additional adjustable parameters, which dilutes the usefulness of the quantified parameters, adds new sources of error, and requires extremely large computational resources. It is the purpose of this work to describe broad-scale transport mechanisms in the brain. Although the simulated concentration contours are “smoothed” by the model assumptions compared to the data, the transport parameters remain valid estimates for each broad region of fluid movement in the brain.

## $T_{1,0}$ Example Image and Histogram

$T_{1,0}$, pre-contrast relaxation time, is dependent on the molecular environment and varies significantly across different biological tissues. As discussed in *Methods*, $T_{1,0}$ can be estimated from baseline MRI signals collected at different flip angles according to the following equation:

$S\left( M_{0}, \alpha\right)= M_{0} sin\left( \alpha\right) \frac{1-e^{\frac{-TR}{T_{1,0}}}}{1-cos\left( \alpha\right)e^{\frac{-TR}{T_{1,0}}}}$ (6)

where:

$TR$ = repetition time (16 ms for the experiments reported here), and

$\alpha$ = flip angle

Shown below are a center sagittal slice of $T_{1,0}$ for a representative mouse (Figure S5), which illustrates different tissues in the mouse brain, and a histogram of $T_{1,0}$ for the same mouse (Figure S6), which shows different groupings of $T_{1,0}$ values. Vasculature has a low $T_{1,0}$, because protons are “refreshed” by the flow of blood, and appears bright in the T1-weighted image. Cerebrospinal fluid in the ventricles has a high $T_{1,0}$ and appears dark in the T1-weighted image.


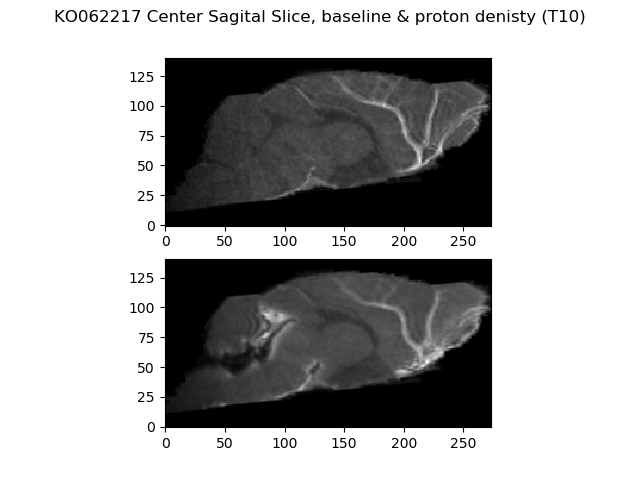


**Figure S5**. Center sagittal image of $T_{1,0}$for mouse KO062217. $T_{1,0}$values correspond to different types of biological tissue. Therefore, different anatomical features can be identified in the MRI images given their calculated $T_{1,0}$. For example, vasculature has a low $T_{1,0}$, because protons are “refreshed” by the flow of blood, and appears bright in the T1-weighted image. Cerebrospinal fluid in the ventricles has a high $T_{1,0}$ and is dark.


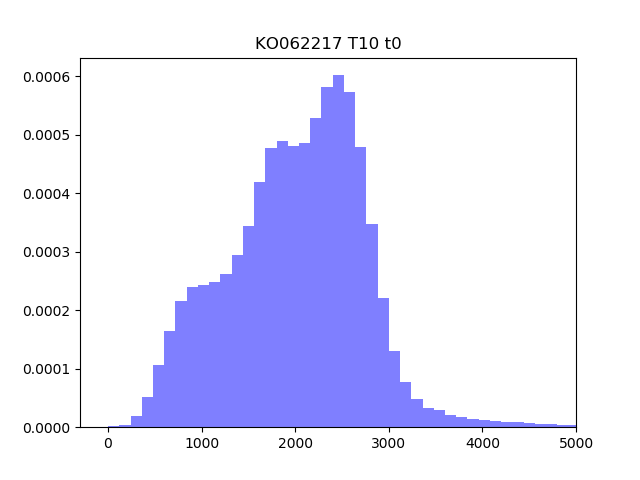


**Figure S6**. Histogram of $T_{1,0}$ for mouse KO062217. $T_{1,0}$values correspond to different types of biological tissue. For example, $T_{1,0}$>3000 corresponds to CSF in the ventricles.

## Relaxivity

Relaxivity of Gadoteridol as a function of magnetic field is reported in Table S1. Although we do not have literature values for the experimental field of 11.75 T, the data show as field strength increases, its effect on relaxivity decreases. Therefore, the lower limit of the range (3.2 L/mmol s) at the highest reported field strength was used in the concentration calculations.

**Table S1**. Gadoteridol (Prohance) Relaxivity in human plasma at different field strengths (5)

| Magnetic Field (Tesla) | 1.5T | 3T | 7T |
| --- | --- | --- | --- |
| Relaxivity (L/mmol s) | 3.8 | 3.3-3.5 | 3.2-3.3 |

## Example Error Contours for determining Optimal Effective Diffusivities

Effective diffusivities were varied and mapped to determine the combination resulting in the minimum root mean square (*rms*) error between the simulation and the data. Root mean square error was summed over all mesh vertices, excluding ventricles, arteries, and T2/T2* interference regions. Error contours for a representative mouse are reported in Figures S7 and S8. A clear minimum is exhibited for $D_{eff,SPAS}$ and $D_{eff,BT}$ (Figure S7), while $D_{eff,PPAS}$ exhibits a significant change in slope, which is deemed the optimal value.


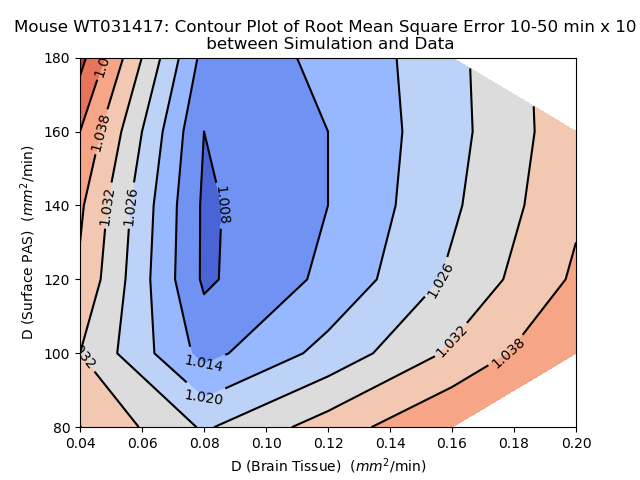


**Figure S7**. Root mean square error times 10 for $D_{eff,SPAS}$ vs. $D_{eff,BT}$ at $D_{eff,PPAS}$=100 mm^2^/min. A clear minimum is exhibited at $D_{eff,BT}$ = 0.08 and $D_{eff,SPAS}$ = 120-140 mm^2^/min.


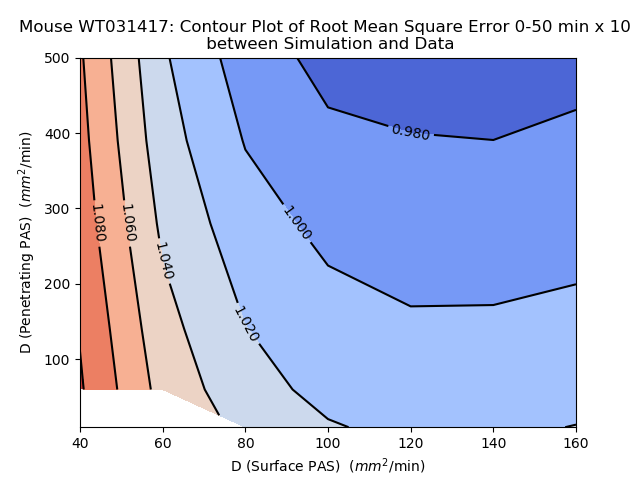


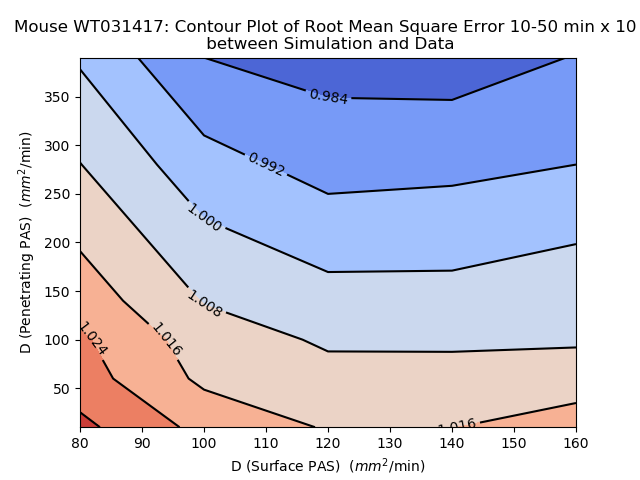


**Figure S8**. Root mean square error times 10 for $D_{eff,PPAS}$ vs. $D_{eff,SPAS}$ at $D_{eff,BT}$ = 0.08 mm^2^/min. $D_{eff,SPAS}$ = 120-140 mm^2^/min as demonstrated in Figure IIA. Surface flattens significantly around $D_{eff,PPAS}$ = 50 mm^2^/min.

1. **Brain Masking and Signal Interference at the Brain Surface**

For the DCE-MRI data used in the analysis, the brain was separated from the rest of the head using the mask shown in Figure S9. The brain mask was generated from a sample mouse dataset and carefully refined by hand, and subsequently registered to each mouse dataset in the study. Note the clear dark boundary surrounding the brain, which is comprised of SAS CSF and skull. Although the two tissues cannot be separated in the T1-weighted image, the SAS is extremely narrow in the murine brain and the boundary is likely to be mostly skull.

**
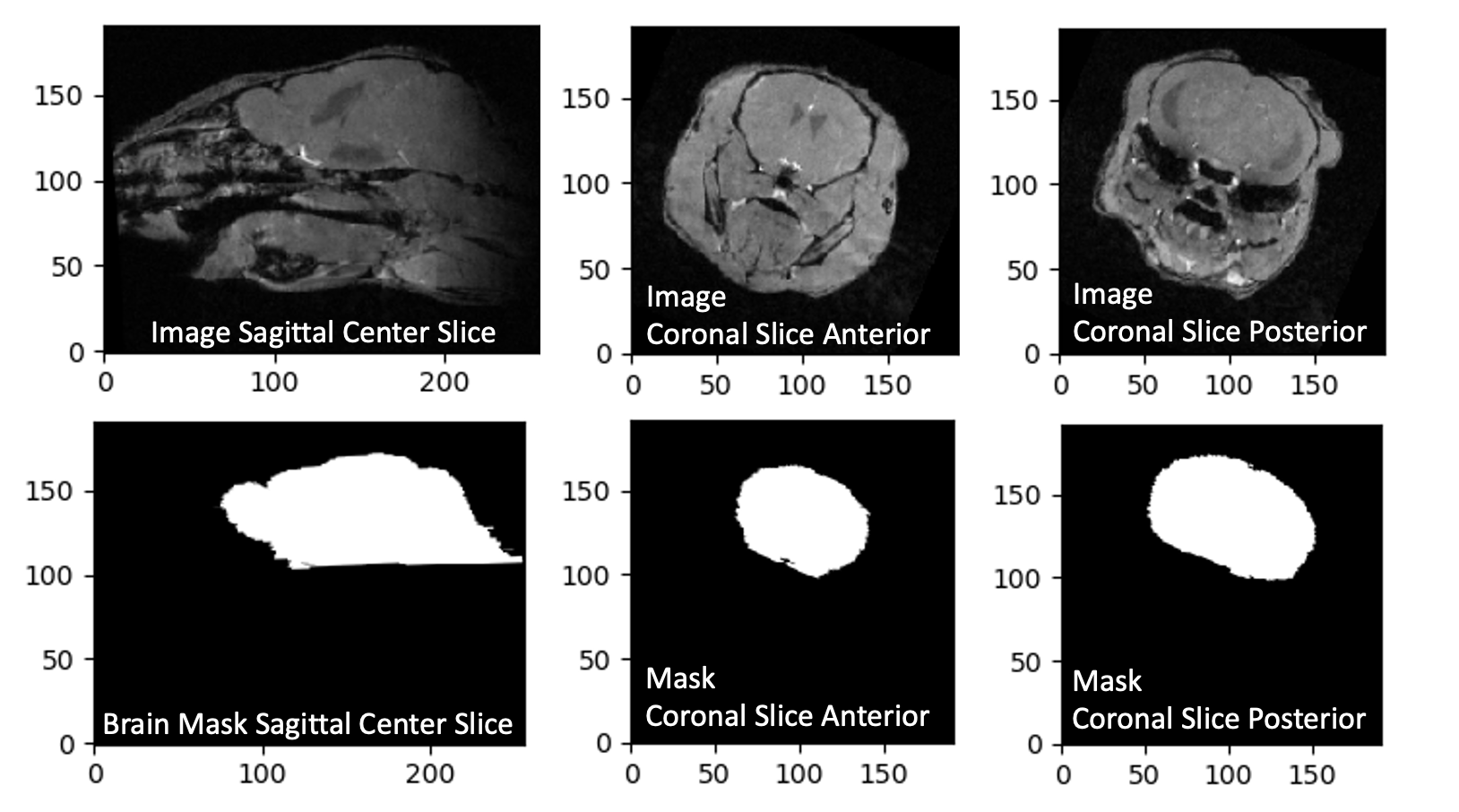
 Figure S9. Brain Mask for MRI Data**. MRI images shown above and brain mask images below. The brain mask was generated from a sample mouse dataset and carefully refined by hand to include brain only, excluding SAS and skull. The CSF in the SAS and the skull are both dark in these T1-weighted MRI images and make a clear barrier. Although the skull and the CSF cannot be separated in this image, the SAS is extremely narrow in the murine brain and likely comprises a small portion of the dark band isolating the brain.

Both signal and calculated concentration images for the masked brain volume exhibit low values at the surface (Figure S10), approximately one to three voxels in thickness. The low surface signal may indicate that some skull tissue and CSF outside the brain, which appear dark in T1-weighted images, was included in the masked volume. However, these surface voxels are also subject to strong local T2/T2* effects from the skull that decrease signal intensities sufficiently to prevent potential contrast enhancement. The surface voxels are most likely comprised of brain tissue and some SAS CSF where signal enhancement from any potential contrast has been obscured by T2/T2* interference.

The most likely source of potential contrast in the surface brain tissue is contrast agent that may have transported from the infusion site through the cisternal aqueduct and to the SAS CSF surrounding the brain. If contrast were present in the SAS CSF surrounding the brain, a concentration gradient would be expected from the brain surface into the brain tissue. Figure S11 shows a graph of concentration converted from DCE-MRI data vs. distance for four instances in the time course moving from the ventral to the dorsal surface of the brain. A significant concentration gradient exists from the ventral surface to around the center of the brain, consistent with a source of contrast in the periarterial space of major arteries. However, from the dorsal surface to the center of the brain, the concentration is near zero with no gradient. If contrast was present in the SAS CSF, a concentration gradient from the surface towards the center of the brain is expected from all surfaces. Given the lack of concentration gradient from the dorsal surface inward, we conclude no contrast agent is present in the SAS CSF. Therefore, our transport analysis is minimally impacted by magnetic susceptibility and errors from “voxel averaging” or tissue mixing at the brain-CSF-skull interface.


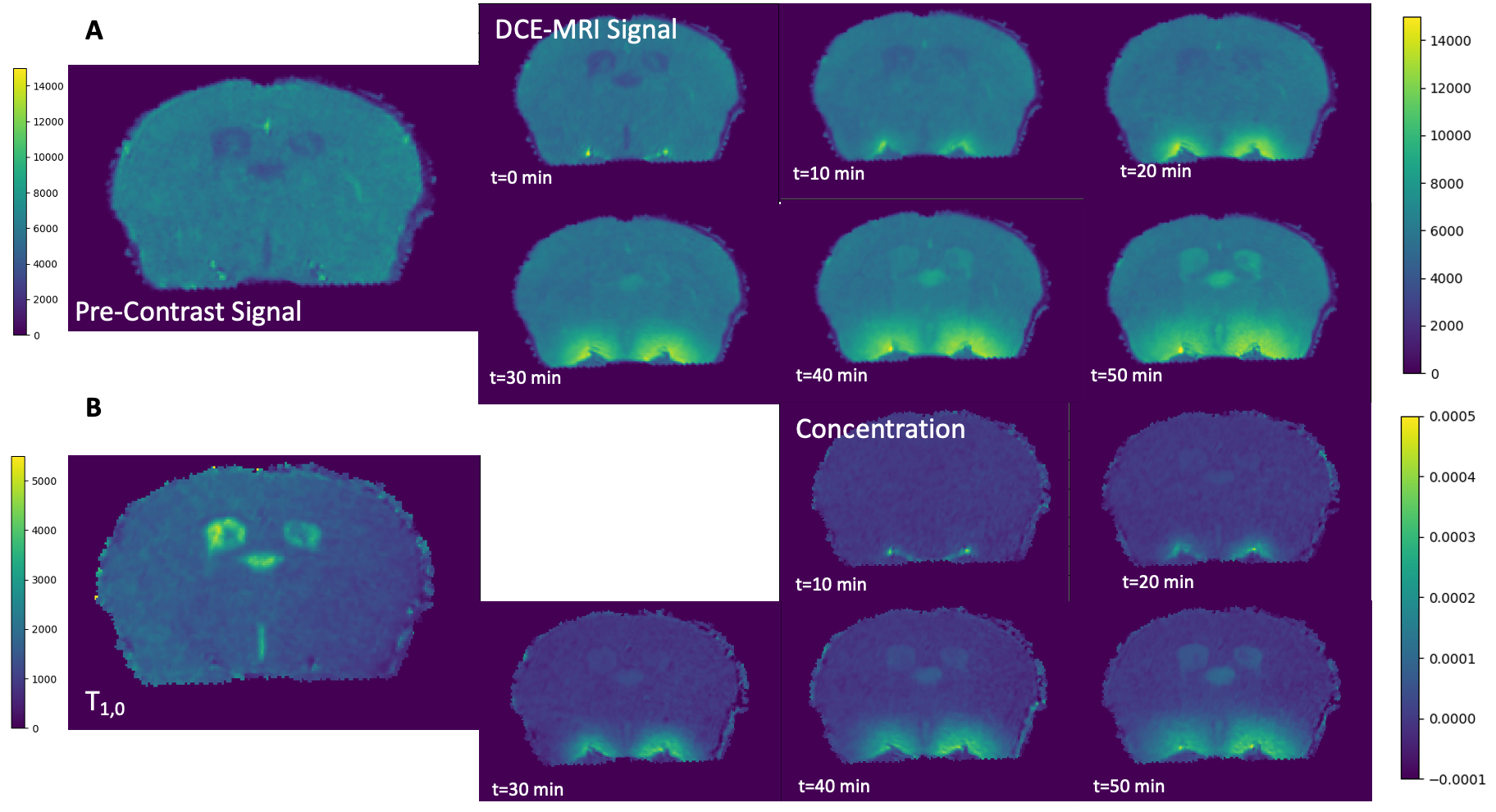


**Figure S10**. **Signal & concentration slices for investigating the brain/skull interface**. **A**. The upper images report a representative coronal slice of DCE-MRI signal, from the pre-contrast image through the first 50 minutes following the initiation of infusion. A layer of low signal is observed at the surface of the brain, which is consistent across the time course. The low signal layer is most likely the result of interference from the strong local T2/T2* signal of the nearby skull that decreases intensities and obscures any potential contrast enhancement in the SAS CSF and surface brain tissue. **B**. The lower images report values calculated from DCE-MRI signal. Furthest left, T_1,0_ is dependent upon the molecular environment and therefore different for different tissues. The surface of the T_1,0_ image reveals localized points with high values aligned with surface arteries and lower values aligned with brain tissue. The remaining images are of contrast concentration and illustrate that concentration at the surface is unchanged throughout the time course. Magnetic susceptibility effects due to skull proximity likely darken intensities at the brain-skull interface sufficiently to prevent potential contrast enhancement.

 **Figure S11.** **Evolution of concentration gradients from the ventral to dorsal surface**. Graphs report concentration calculated from DEC-MRI data vs. distance for four instances in the time course for the orange lines depicted in the central image moving from the ventral to the dorsal surface of the brain. The central image is a coronal slice of contrast concentration calculated from DCE-MRI signal at t=50 min. The graphs show high concentration near the ventral surface decreasing as one moves towards the dorsal surface. Near the center of the brain, contrast concentration is near zero and the concentration curve becomes flat. The concentration near the ventral surface increases in time and the spatial trend remains similar. The concentration curves are indicative of a source of contrast near the ventral surface, periarterial space of major arteries, and no contrast source at the dorsal surface. If contrast was present in the SAS CSF, it would surround the brain resulting in concentration gradients from all surfaces.

## **Periarterial Space width Sensitivity Analysis and Periarterial Space as Preferential Transport Routes**

The periarterial spaces were identified based on their unique concentration dynamics and are named periarterial due to their position, surrounding the major arteries. Periarterial space in the segmented model does not necessarily denote anatomical periarterial space. In this supplementary section, we investigate 1) the optimal width of the periarterial subdomain in the model that best represents the DCE-MRI concentration data and 2) how the periarterial space segmented for the model compares to current measurements of perivascular transport routes characterized by Mestre et al. (6) and Tithof et al. (7).

- 1. **Periarterial Space Width Sensitivity Analysis**

To segment a surface periarterial space (PAS_Surf_) subdomain that best represents the concentration data, a sensitivity analysis was performed to determine the PAS_Surf_ width that resulted in the minimum ***rms*** error, or difference between the simulation and the data. The analysis was performed at BT $D_{eff}$=0.2 and PAS_Surf_ $D_{eff}$=72 mm^2^/min without segmentation of the branching periarterial space for simplicity. A ***rms*** minimum was observed around 5,500 total mesh vertices in the PAS_Surf_ region (Figure S12). (Total vertices greater than 6,400 were not investigated because such regions deviated too far outside the marked change in contrast agent concentration that delineated the transport region.) The number of PAS_Surf_ vertices is directly proportional to the average width of the periarterial space, where 5,500 vertices correspond to a PAS_Surf_ width of around 400 $\mu$m at the Circle of Willis.


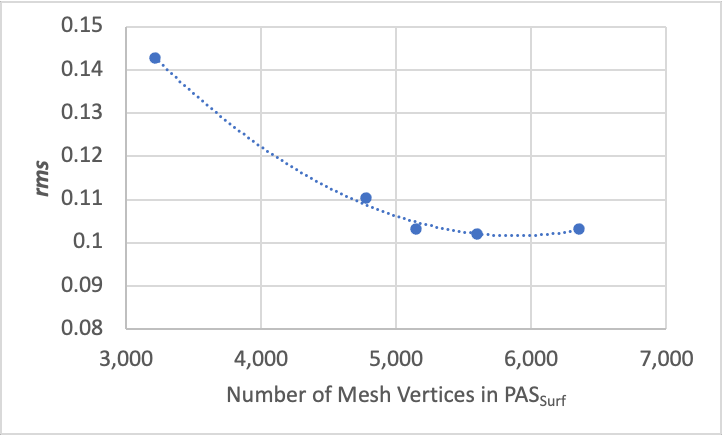


**Figure S12**. Results of sensitivity analysis for ***rms*** error vs. total number of vertices in the surface periarterial space (PAS_Surf_) subdomain. The number of PAS_Surf_ vertices is directly proportional to the average width of the periarterial space. A minimum is exhibited around 5,500 vertices which corresponds to a PAS_Surf_ width of around 400 $\mu$m at the Circle of Willis.

- 1. **Preferential Transport Routes correspond to Periarterial Space**

The model subdomains surrounding the arteries (the PAS_Surf_ and PAS_Branch_) were defined by their unique contrast agent concentration and transport dynamics, not by MRI-measured tissue properties (notably *T_1,0_*) normal used to identify anatomical features. Based on the sensitivity analysis performed above, the DCE-MRI data indicate that the determined boundaries of the PAS_Surf_ subdomain are significant to transport. From brain anatomy and the glymphatic model (Figure 1) discussed in the *Introduction*, these segmented volumes, which follow and surround the major arteries of each subject (Figure S2), appear to correlate with anatomical periarterial space (PAS).

Using *in vivo* particle tracking velocimetry and imaging, Mestre et al. found the width of the preferential transport routes, identified by the authors as periarterial space, to be similar to the diameter of the vessel it surrounds (6)--observed PAS widths were 0.75 to 1.5 times the artery diameter (Figure 1e in Mestre et al.). This study also demonstrated that fixation methods used for *in vitro* imaging cause collapse of the PAS leading to substantial shrinkage and bringing into question previous *in vitro* measurements of a narrower PAS. Tithof et al. measured PAS dimensions *in vivo*, determining the PAS width for surface arteries (at its widest point) to be 1 to 2.5 times the artery diameter (7). For example, the internal carotid artery (ICA), which is part of the Circle of Willis, has a diameter of around 140 μm (measured *ex vivo*, which may cause shrinkage) (8). Therefore, one would estimate a PAS width at 105 – 350 μm.

The width of the PAS_Surf_ in the transport model, measured close to the ICA, averages 400 + 100 μm--larger than the highest estimate from *in vivo* imaging. However, the dimensions of the segmented subdomains are impacted by artifacts inherent to MRI and by interpolation of data onto the finite-element mesh. The MRI signal for each voxel is the average of the materials inside the voxel. So, if an anatomical feature exists within two neighboring voxels, it will have an impact on the signal for both voxels. The registered voxel width for the experiments reported here is 60 μm. Thus, a feature of dimension 60 μm that “straddles” two voxels will have an apparent width of 120 μm, a stretch factor of 2 or greater. Near the vasculature, this phenomenon is amplified by the pulsing of the arteries, which causes movement of the artery and the surrounding tissue (the periarterial space) relative to the MRI grid and blurs neighboring voxels. For example, the large branching arteries (OA, PCA, and MCA) are known to have a diameter around 40-90 μm (9). Within the MRI data, these arteries have a diameter of about 3 voxels or 180 μm, a stretch factor of 2 to 4. In the finite-element model, their diameter increases to about 220 μm, a 20% increase from the MRI data, due to interpolation of the MRI data onto the less-refined finite-element mesh. Therefore, both spread and blur attributed to the experimental and modelling techniques account for a stretching of the PAS by 2 to 4 times, corresponding to a PAS width of 280 – 560 μm near the ICA and bringing the width of the PAS_Surf_ and surface periarterial space, as characterized by *in vivo* studies, into agreement.

In conclusion, the subdomains defined by the model surrounding the arteries (PAS_Surf_ and PAS_Branch_) correlate well with the dimensions of the preferential transport routes characterized in the literature and identified as periarterial space (6, 7). Artifacts inherent to MRI and interpolation cause the periarterial space defined by the DCE-MRI data and transport model to be stretched relative to its actual dimensions.

## Example FEniCS Code for Whole-Brain Finite-Element Model

# The purpose of this code is to model transport in the mouse brain.

# In particular effective diffusivities for specific regions in the brain

# are varied and compared to DCE MRI data to determine the set of

# effective diffusivities that minimizes the difference, or rms error.

# The domain surface was extracted from MRI data using MRIcrOS

# The domain was meshed using gmsh and this mesh is imported in this code.

# Anatomical subdomains were defined using thresholds for T1,0 or conc

# and were then interpolated onto the mesh. Each anatomical mask is imported

# and then used to create subdomains with unique effective diffusivities.

# the data has been interpolated onto the mesh so rms can be directly calculated.

# FEM problem is solved using FEniCS project (dolfin and mshr).

from dolfin import *

from mshr import *

import numpy as np

#----Inputs--------------------------------------------------------------------

# Enter Effective Diffusivities to be Tested

DBT = np.zeros(3)

DSPAS = np.zeros(4)

DPPAS = np.zeros(1)

len_DBT = DBT.shape[0]

len_DSPAS = DSPAS.shape[0]

len_DPPAS = DPPAS.shape[0]

DBT[0] = 0.08

DBT[1] = 0.04

DBT[2] = 0.12

DSPAS[0] = 160

DSPAS[1] = 140

DSPAS[2] = 120

DSPAS[3] = 100

DPPAS[0] = 100

print(DBT, DSPAS, DPPAS)

# Define Parameters

time_pts = 8

data_dt = 10.4 #min

Q = 0.00335 # source rate = 66.7 mM * 0.5 e-6 l/min = 0.0000335x100

src = Point(-0.09, -4.89, -0.78) # source location

dt = data_dt / 10.

T = (time_pts) * data_dt

#----Various Error Functions---------------------------------------------------

def sim_rms(original, sim):

rms = np.sqrt(np.mean((original-sim)**2))

rmspe = np.sqrt(np.mean(np.square((original-sim)/original)))

return (rms, rmspe)

def mape(original, sim):

mpe = np.mean((original-sim)/original)

mape = np.mean(np.abs((original-sim)/original))

return (mape, mpe)

#----Import Data---------------------------------------------------------------

# Load concentration data

conc_0 = np.load('mouse_interpconct0ext_ondomain_t1.npy')

conc_1 = np.load('mouse_interpconct0ext_ondomain_t2.npy')

conc_2 = np.load('mouse_interpconct0ext_ondomain_t3.npy')

conc_3 = np.load('mouse_interpconct0ext_ondomain_t4.npy')

conc_4 = np.load('mouse_interpconct0ext_ondomain_t5.npy')

conc_5 = np.load('mouse_interpconct0ext_ondomain_t6.npy')

conc_6 = np.load('mouse_interpconct0ext_ondomain_t7.npy')

conc_7 = np.load('mouse_interpconct0ext_ondomain_t8.npy')

# combine into one array

conc_m = np.concatenate((conc_0, conc_1, conc_2, conc_3, conc_4, conc_5, conc_6, conc_7), axis=1)

# Load T2 mask data

# T2 interference has rendered data in these voxels useless and they are

#excluded from the error calculations. T2 interference region changes in time

T2mask_0 = np.load('mouse_interpT2ext2mask_t1.npy')

T2mask_1 = np.load('mouse_interpT2ext2mask_t2.npy')

T2mask_2 = np.load('mouse_interpT2ext2mask_t3.npy')

T2mask_3 = np.load('mouse_interpT2ext2mask_t4.npy')

T2mask_4 = np.load('mouse_interpT2ext2mask_t5.npy')

T2mask_5 = np.load('mouse_interpT2ext2mask_t6.npy')

T2mask_6 = np.load('mouse_interpT2ext2mask_t7.npy')

T2mask_7 = np.load('mouse_interpT2ext2mask_t8.npy')

# combine into one array

T2_mask = np.concatenate((T2mask_0, T2mask_1, T2mask_2, T2mask_3, T2mask_4, T2mask_5, T2mask_6, T2mask_7), axis=1)

print(T2_mask.shape)

# load anatomical mask data

# ventricles

vent_mask = np.load('Mouse_vent_interp_00706.npy')

# arteries

Acer_mask = np.load('Mouse_interp_acer_nofilt.npy')

aCoW_mask = np.load('Mouse_interp_surfart_nofilt.npy')

Bas_mask = np.load('Mouse_interp_bas_nofilt.npy')

Pcer_mask = np.load('Mouse_interp_pcermask_nofilt.npy')

Mcer_mask = np.load('Mouse_interp_mcermask_nofilt.npy')

Olfac_mask = np.load('Mouse_interp_olfacmask_nofilt.npy')

# periarterial space

CoW_mask = np.load('Mouse_interpt0_SPASrev0924b_01.npy') # surface arteries

penperi_mask = np.load('Mouse_interp_PPAS_rev1011cp_01.npy') # branching arteries

# Load the Mesh

mesh = Mesh('mouse_mesh_00706.xml.gz')

coor = mesh.coordinates()

# save mesh

mfilename = 'mouse_mesh_00706.pvd'

meshfile = File(mfilename)

meshfile << mesh

#----exclude certain vertices from error calculation---------------------------

# ventricle points

nv = mesh.num_vertices()

exclude = np.zeros((nv, time_pts))

for i in range(nv):

if (vent_mask[i] > 0 or aCoW_mask[i] > 0 or Bas_mask[i] > 0):

for j in range(time_pts):

exclude[i, j] = 1

# conc_m=0 points

for i in range(nv):

for j in range(time_pts):

if conc_m[i,j] == 0:

exclude[i, j] = 1

# T2 interference points

for i in range(nv):

for j in range(time_pts):

if T2_mask[i, j] > 0:

exclude[i, j] = 1

#---Map concentration data onto mesh for erro calc-----------------------------

# create Function space

C = FunctionSpace(mesh, 'Lagrange', 2)

comm = MPI.comm_world

cncdatafile = HDF5File(comm, 'mouse_concdata_T2ext.h5', 'r')

# map concentration data onto function space

CD = FunctionSpace(mesh, 'CG', 1)

cd1 = Function(CD)

cncdatafile.read(cd1, '/cd1')

cd2 = Function(CD)

cncdatafile.read(cd2, '/cd2')

cd3 = Function(CD)

cncdatafile.read(cd3, '/cd3')

cd4 = Function(CD)

cncdatafile.read(cd4, '/cd4')

cd5 = Function(CD)

cncdatafile.read(cd5, '/cd5')

cd6 = Function(CD)

cncdatafile.read(cd6, '/cd6')

cd7 = Function(CD)

cncdatafile.read(cd7, '/cd7')

cd8 = Function(CD)

cncdatafile.read(cd8, '/cd8')

#----Create anatomical subdomains----------------------------------------------

# create anatomical arrays for use in marking subdomains

vertex_values = np.zeros(mesh.num_vertices())

vent_vertex_values = np.zeros(mesh.num_vertices())

Acer_vertex_values = np.zeros(mesh.num_vertices())

SA_vertex_values = np.zeros(mesh.num_vertices())

Bas_vertex_values = np.zeros(mesh.num_vertices())

Mcer_vertex_values = np.zeros(mesh.num_vertices())

Pcer_vertex_values = np.zeros(mesh.num_vertices())

Olf_vertex_values = np.zeros(mesh.num_vertices())

CoW_vertex_values = np.zeros(mesh.num_vertices())

PP_vertex_values = np.zeros(mesh.num_vertices())

# map anatomical masks onto mesh using a Function

# vertex to dof_map approach

AN = FunctionSpace(mesh, 'CG', 1) # so that dofs are only in mesh vertices

# ventricles

vm = Function(AN)

for vertex in vertices(mesh):

vent_vertex_values[vertex.index()] = vent_mask[vertex.index()]

vm.vector()[:] = vent_vertex_values[dof_to_vertex_map(AN)]

# arteries

SAm = Function(AN)

SAm.set_allow_extrapolation(True) #work around--rounding error causes crash

for vertex in vertices(mesh):

SA_vertex_values[vertex.index()] = aCoW_mask[vertex.index()]

SAm.vector()[:] = SA_vertex_values[dof_to_vertex_map(AN)]

Acerm = Function(AN)

Acerm.set_allow_extrapolation(True) #work around--rounding error causes crash

for vertex in vertices(mesh):

Acer_vertex_values[vertex.index()] = Acer_mask[vertex.index()]

Acerm.vector()[:] = Acer_vertex_values[dof_to_vertex_map(AN)]

Basm = Function(AN)

Basm.set_allow_extrapolation(True) #work around--rounding error causes crash

for vertex in vertices(mesh):

Bas_vertex_values[vertex.index()] = Bas_mask[vertex.index()]

Basm.vector()[:] = Bas_vertex_values[dof_to_vertex_map(AN)]

# periarterial space

CoWm = Function(AN)

CoWm.set_allow_extrapolation(True) #work around--rounding error causes crash

for vertex in vertices(mesh):

CoW_vertex_values[vertex.index()] = CoW_mask[vertex.index()]

CoWm.vector()[:] = CoW_vertex_values[dof_to_vertex_map(AN)]

PPm = Function(AN)

PPm.set_allow_extrapolation(True) #work around--rounding error causes crash

for vertex in vertices(mesh):

PP_vertex_values[vertex.index()] = penperi_mask[vertex.index()]

PPm.vector()[:] = PP_vertex_values[dof_to_vertex_map(AN)]

# more arteries

Mcerm = Function(AN)

Mcerm.set_allow_extrapolation(True) #work around--rounding error causes crash

for vertex in vertices(mesh):

Mcer_vertex_values[vertex.index()] = Mcer_mask[vertex.index()]

Mcerm.vector()[:] = Mcer_vertex_values[dof_to_vertex_map(AN)]

Pcerm = Function(AN)

Pcerm.set_allow_extrapolation(True) #work around--rounding error causes crash

for vertex in vertices(mesh):

Pcer_vertex_values[vertex.index()] = Pcer_mask[vertex.index()]

Pcerm.vector()[:] = Pcer_vertex_values[dof_to_vertex_map(AN)]

Olfm = Function(AN)

Olfm.set_allow_extrapolation(True) #work around--rounding error causes crash

for vertex in vertices(mesh):

Olf_vertex_values[vertex.index()] = Olfac_mask[vertex.index()]

Olfm.vector()[:] = Olf_vertex_values[dof_to_vertex_map(AN)]

# mark anatomical subdomaons

materials = MeshFunction("size_t", mesh, mesh.topology().dim())

materials.set_all(0)

class Ventricle(SubDomain):

def inside(self, x, on_boundary):

if vm(x[0], x[1], x[2]) > 0:

return True

else:

return False

subdomain1 = Ventricle()

subdomain1.mark(materials, 1)

class SA(SubDomain):

def inside(self, x, on_boundary):

if SAm(x[0], x[1], x[2]) > 0:

return True

else:

return False

subdomain4 = SA()

subdomain4.mark(materials, 1)

class Acer(SubDomain):

def inside(self, x, on_boundary):

if Acerm(x[0], x[1], x[2]) > 0:

return True

else:

return False

subdomain5 = Acer()

subdomain5.mark(materials, 1)

class Bas(SubDomain):

def inside(self, x, on_boundary):

if Basm(x[0], x[1], x[2]) > 0:

return True

else:

return False

subdomain6 = Bas()

subdomain6.mark(materials, 1)

class PenPeri(SubDomain):

def inside(self, x, on_boundary):

if PPm(x[0], x[1], x[2]) > 0:

return True

else:

return False

subdomain3 = PenPeri()

subdomain3.mark(materials, 3)

class Mcer(SubDomain):

def inside(self, x, on_boundary):

if Mcerm(x[0], x[1], x[2]) > 0:

return True

else:

return False

subdomain7 = Mcer()

subdomain7.mark(materials, 1)

class Pcer(SubDomain):

def inside(self, x, on_boundary):

if Pcerm(x[0], x[1], x[2]) > 0:

return True

else:

return False

subdomain8 = Pcer()

subdomain8.mark(materials, 1)

class Olf(SubDomain):

def inside(self, x, on_boundary):

if Olfm(x[0], x[1], x[2]) > 0:

return True

else:

return False

subdomain9 = Olf()

subdomain9.mark(materials, 1)

class CoW(SubDomain):

def inside(self, x, on_boundary):

if CoWm(x[0], x[1], x[2]) > 0:

return True

else:

return False

subdomain2 = CoW()

subdomain2.mark(materials, 2)

# save subdomains to file

mfilename = 'sim_data_WT031417/mouse_matls_comp2_rev0924b01_rev1011c01_ppnv.pvd'

matlsfile = File(mfilename)

matlsfile << materials

#----Begin loop generating conc simulations for various effective diffusivities

err=np.zeros((len_DBT*len_DSPAS*len_DPPAS, 15))

err_ct = 0

for k in range(len_DBT):

for l in range(len_DSPAS):

for m in range(len_DPPAS):

d_0 = DBT[k]

d_1 = d_0 * 0.00000001

d_2 = DSPAS[l]

d_3 = DPPAS[m]

print('Diffusivities: ', d_0, d_2, d_3)

# create files to save simulation concentration

cfilename = 'Map/WT031417_concl_D'+str(d_0)+'Q100_SPAS'+str(d_2)+'_PPAS'+str(d_3)+'.pvd'

cncfile = File(cfilename)

# and difference between simulation and data for visualization

delfilename = 'Map/WT031417_deltal_D'+str(d_0)+'Q100_SPAS'+str(d_2)+'_PPAS'+str(d_3)+'..pvd'

delfile = File(delfilename)

#----define diffusivity on subdomains------------------------------------------

class Diff(UserExpression):

def __init__(self, materials, d_0, d_1, d_2, d_3, **kwargs):

super().__init__(**kwargs)

self.materials = materials

self.d_0 = d_0

self.d_1 = d_1

self.d_2 = d_2

self.d_3 = d_3

def eval_cell(self, values, x, cell):

if self.materials[cell.index] == 1:

values[0] = self.d_1

elif self.materials[cell.index] == 2:

values[0] = self.d_2

elif self.materials[cell.index] == 3:

values[0] = self.d_3

else:

values[0] = self.d_0

def value_shape(self):

return ()

AD = Diff(materials, d_0, d_1, d_2, d_3, degree=0)

# mark boundaries

def boundary(x, on_boundary):

return on_boundary

# define boundary condition

# no flux boundary condition is the natural boundary condition 0*ds

# define initial condition

c_1 = interpolate(Constant(0.0), C)

#----define finite element variational problem---------------------------------

c = TrialFunction(C)

phi = TestFunction(C)

a = phi*c*dx + dt*AD*inner(nabla_grad(phi), nabla_grad(c))*dx

L = c_1*phi*dx

A = assemble(a)

b = assemble(L)

# solve concentration problem

# define functions

c = Function(C)

# Transfer a function in CD to a function in C using a transfer matrix, M:

# used for calculating difference between simulation and data for visualization

M = PETScDMCollection.create_transfer_matrix(CD,C)

cdc = Function(C)

delta = Function(C)

t = dt

time_pt = 0

conc = np.zeros((nv, time_pts))

#---time stepping loop, solve dynamic mass transport PDE-----------------------

while t <=T+.0000000001:

b = assemble(L, tensor=b)

# add or remove point source

source_add = PointSource(C, src, Q)

source_add.apply(b)

if t >= 20.:

source_remove = PointSource(C, src, -Q)

source_remove.apply(b)

# bc.apply(A, b)

solve(A, c.vector(), b, "gmres", "ilu")

# save concentration sim to array and to file at data time points

if (t<10.5 and t>10.3):

#save to file

cncfile << c, t

cdc.vector()[:] = M*cd1.vector()

delta.vector()[:] = cdc.vector()[:]

delta.vector()[:] = delta.vector()[:] - c.vector()[:]

delfile << delta, t

# save to array for error cacluation at end

vertex_values_c = c.compute_vertex_values(mesh)

conc[:, time_pt] = vertex_values_c[:]

time_pt += 1

# new_bc = 'cd' + str(time_pt)

# cb.assign(new_bc)

if (t<20.9 and t>20.7):

cncfile << c, t

cdc.vector()[:] = M*cd2.vector()

delta.vector()[:] = cdc.vector()[:]

delta.vector()[:] = delta.vector()[:] - c.vector()[:]

delfile << delta, t

vertex_values_c = c.compute_vertex_values(mesh)

conc[:, time_pt] = vertex_values_c[:]

time_pt += 1

if (t<31.3 and t>31.1):

cncfile << c, t

cdc.vector()[:] = M*cd3.vector()

delta.vector()[:] = cdc.vector()[:]

delta.vector()[:] = delta.vector()[:] - c.vector()[:]

delfile << delta, t

vertex_values_c = c.compute_vertex_values(mesh)

conc[:, time_pt] = vertex_values_c[:]

time_pt += 1

if (t<41.7 and t>41.5):

cncfile << c, t

cdc.vector()[:] = M*cd4.vector()

delta.vector()[:] = cdc.vector()[:]

delta.vector()[:] = delta.vector()[:] - c.vector()[:]

delfile << delta, t

vertex_values_c = c.compute_vertex_values(mesh)

conc[:, time_pt] = vertex_values_c[:]

time_pt += 1

if (t<52.1 and t>51.9):

cncfile << c, t

cdc.vector()[:] = M*cd5.vector()

delta.vector()[:] = cdc.vector()[:]

delta.vector()[:] = delta.vector()[:] - c.vector()[:]

delfile << delta, t

vertex_values_c = c.compute_vertex_values(mesh)

conc[:, time_pt] = vertex_values_c[:]

time_pt += 1

if (t<62.5 and t>62.3):

cncfile << c, t

cdc.vector()[:] = M*cd6.vector()

delta.vector()[:] = cdc.vector()[:]

delta.vector()[:] = delta.vector()[:] - c.vector()[:]

delfile << delta, t

vertex_values_c = c.compute_vertex_values(mesh)

conc[:, time_pt] = vertex_values_c[:]

time_pt += 1

if (t<72.9 and t>72.7):

cncfile << c, t

cdc.vector()[:] = M*cd7.vector()

delta.vector()[:] = cdc.vector()[:]

delta.vector()[:] = delta.vector()[:] - c.vector()[:]

delfile << delta, t

vertex_values_c = c.compute_vertex_values(mesh)

conc[:, time_pt] = vertex_values_c[:]

time_pt += 1

if (t<83.3 and t>83.1):

cncfile << c, t

cdc.vector()[:] = M*cd8.vector()

delta.vector()[:] = cdc.vector()[:]

delta.vector()[:] = delta.vector()[:] - c.vector()[:]

delfile << delta, t

vertex_values_c = c.compute_vertex_values(mesh)

conc[:, time_pt] = vertex_values_c[:]

time_pt += 1

c_1.assign(c)

t += dt

#---calculate fit--------------------------------------------------------------

for i in range(nv):

for j in range(time_pts):

if exclude[i, j] > 0:

conc[i, j] = 1.

conc_m[i, j] = 1.

err[err_ct, 0] = d_0

err[err_ct, 1] = d_2

err[err_ct, 2] = d_3

print('Diffusivities: ', d_0, d_2, d_3)

err_mape, err_mpe = mape(conc_m[:,0:4], conc[:,0:4])

err_rms, err_rmspe = sim_rms(conc_m[:,0:4], conc[:,0:4])

err[err_ct, 3] = err_rms

err[err_ct, 4] = err_rmspe

err[err_ct, 5] = err_mape

print('total exclude T2 error--t=10-50 min')

print('exclude T2 mape = ', err_mape)

print('exclude T2 rms = ', err_rms)

print('exclude T2 rmspe = ', err_rmspe)

err_mape, err_mpe = mape(conc_m[:,0:3], conc[:,0:3])

err_rms, err_rmspe = sim_rms(conc_m[:,0:3], conc[:,0:3])

err[err_ct, 6] = err_rms

err[err_ct, 7] = err_rmspe

err[err_ct, 8] = err_mape

print('total exclude T2 error--t=10-40 min')

print('exclude T2 mape = ', err_mape)

print('exclude T2 rms = ', err_rms)

print('exclude T2 rmspe = ', err_rmspe)

err_mape, err_mpe = mape(conc_m[:,0:2], conc[:,0:2])

err_rms, err_rmspe = sim_rms(conc_m[:,0:2], conc[:,0:2])

err[err_ct, 9] = err_rms

err[err_ct, 10] = err_rmspe

err[err_ct, 11] = err_mape

print('total exclude T2 error--t=10-30 min')

print('exclude T2 mape = ', err_mape)

print('exclude T2 rms = ', err_rms)

print('exclude T2 rmspe = ', err_rmspe)

err_mape, err_mpe = mape(conc_m[:,0:7], conc[:,0:7])

err_rms, err_rmspe = sim_rms(conc_m[:,0:7], conc[:,0:7])

err[err_ct, 12] = err_rms

err[err_ct, 13] = err_rmspe

err[err_ct, 14] = err_mape

print('total exclude T2 error--t=10-80 min')

print('exclude T2 mape = ', err_mape)

print('exclude T2 rms = ', err_rms)

print('exclude T2 rmspe = ', err_rmspe)

err_ct += 1

np.save('Map/WT031417_errcont_030521.npy', err)

**References:**

1. Iliff JJ, Lee H, Yu M, Feng T, Logan J, Nedergaard M, et al. Brain-wide pathway for waste clearance captured by contrast-enhanced MRI. Journal of Clinical Investigation. 2013;123(3):1299-309. DOI: 10.1172/jci67677.

2. Jiang Q, Zhang L, Ding GL, Davoodi-Bojd E, Li QJ, Li L, et al. Impairment of the glymphatic system after diabetes. J Cereb Blood Flow Metab. 2017;37(4):1326-37. DOI: 10.1177/0271678x16654702.

3. Zhou Y, Cai JS, Zhang WH, Gong XX, Yan SQ, Zhang KM, et al. Impairment of the Glymphatic Pathway and Putative Meningeal Lymphatic Vessels in the Aging Human. Annals of Neurology. 2020;87(3):357-69. DOI: 10.1002/ana.25670.

4. Benveniste H, Heerdt PM, Fontes M, Rothman DL, Volkow ND. Glymphatic System Function in Relation to Anesthesia and Sleep States. Anesthesia and Analgesia. 2019;128(4):747-58. DOI: 10.1213/ane.0000000000004069.

5. Szomolanyi P, Rohrer M, Frenzel T, Noebauer-Hohmann IM, Jost G, Endrikat J, et al. Comparison of the Relaxivities of Macrocyclic Gadolinium-Based Contrast Agents in Human Plasma at 1.5, 3, and 7 T, and Blood at 3 T. Investigative Radiology. 2019;54(9):559-64. DOI: 10.1097/rli.0000000000000577.

6. Mestre H, Tithof J, Du T, Song W, Peng WG, Sweeney AM, et al. Flow of cerebrospinal fluid is driven by arterial pulsations and is reduced in hypertension. Nature Communications. 2018;9. DOI: 10.1038/s41467-018-07318-3.

7. Tithof J, Kelley DH, Mestre H, Nedergaard M, Thomas JH. Hydraulic resistance of periarterial spaces in the brain. Fluids and Barriers of the Cns. 2019;16. DOI: 10.1186/s12987-019-0140-y.

8. Qian B, Rudy RF, Cai T, Du R. Cerebral Artery Diameter in Inbred Mice Varies as a Function of Strain. Frontiers in neuroanatomy. 2018;12:10-. DOI: 10.3389/fnana.2018.00010.

9. Xiong B, Li A, Lou Y, Chen S, Long B, Peng J, et al. Precise Cerebral Vascular Atlas in Stereotaxic Coordinates of Whole Mouse Brain. Frontiers in Neuroanatomy. 2017;11. DOI: 10.3389/fnana.2017.00128.
